# Effects of irrigation on root growth and development of soybean: A 3-year sandy field experiment

**DOI:** 10.3389/fpls.2022.1047563

**Published:** 2022-12-14

**Authors:** Khuynh The Bui, Toshiya Naruse, Hideki Yoshida, Yusuke Toda, Yoshihiro Omori, Mai Tsuda, Akito Kaga, Yuji Yamasaki, Hisashi Tsujimoto, Yasunori Ichihashi, Masami Hirai, Toru Fujiwara, Hiroyoshi Iwata, Makoto Matsuoka, Hirokazu Takahashi, Mikio Nakazono

**Affiliations:** ^1^ Graduate School of Bio-Agricultural Sciences, Nagoya University, Nagoya, Japan; ^2^ Faculty of Agronomy, Vietnam National University of Agriculture, Hanoi, Vietnam; ^3^ Bioscience and Biotechnology Center, Nagoya University, Nagoya, Japan; ^4^ Graduate School of Agricultural and Life Sciences, The University of Tokyo, Tokyo, Japan; ^5^ Institute for Agro-Environmental Sciences, National Agriculture and Food Research Organization (NARO), Ibaraki, Japan; ^6^ Tsukuba-Plant Innovation Research Center (T-PIRC), University of Tsukuba, Tsukuba, Japan; ^7^ Institute of Crop Science, National Agriculture and Food Research Organization (NARO), Tsukuba, Japan; ^8^ Arid Land Research Center, Tottori University, Tottori, Japan; ^9^ RIKEN BioResource Research Center (BRC), Tsukuba, Japan; ^10^ RIKEN Center for Sustainable Resource Science, Tsukuba, Japan; ^11^ School of Plant Biology, The University of Western Australia, 35 Stirling Highway, Crawley, WA, Australia

**Keywords:** irrigation, root growth, stability, field phenotyping, genetic diversity

## Abstract

Increasing the water use efficiency of crops is an important agricultural goal closely related to the root system —the primary plant organ for water and nutrient acquisition. In an attempt to evaluate the response of root growth and development of soybean to water supply levels, 200 genotypes were grown in a sandy field for 3 years under irrigated and non-irrigated conditions, and 14 root traits together with shoot fresh weight and plant height were investigated. Three-way ANOVA revealed a significant effect of treatments and years on growth of plants, accounting for more than 80% of the total variability. The response of roots to irrigation was consistent over the years as most root traits were improved by irrigation. However, the actual values varied between years because the growth of plants was largely affected by the field microclimatic conditions (i.e., temperature, sunshine duration, and precipitation). Therefore, the best linear unbiased prediction values for each trait were calculated using the original data. Principal component analysis showed that most traits contributed to principal component (PC) 1, whereas average diameter, the ratio of thin and medium thickness root length to total root length contributed to PC2. Subsequently, we focused on selecting genotypes that exhibited significant improvements in root traits under irrigation than under non-irrigated conditions using the increment (I-index) and relative increment (RI-index) indices calculated for all traits. Finally, we screened for genotypes with high stability and root growth over the 3 years using the multi-trait selection index (MTSI).Six genotypes namely, GmJMC130, GmWMC178, GmJMC092, GmJMC068, GmWMC075, and GmJMC081 from the top 10% of genotypes scoring MTSI less than the selection threshold of 7.04 and 4.11 under irrigated and non-irrigated conditions, respectively, were selected. The selected genotypes have great potential for breeding cultivars with improved water usage abilities, meeting the goal of water-saving agriculture.

## 1 Introduction

Agriculture is highly water-demanding and accounts for about 70% of total water usage (Ritchie and Roser, 2018) in an era where climate change threatens crop production worldwide (Calleja-Cabrera et al., 2020). To feed the increasing population by 2050, an additional 44 million tons of food will be required annually (Tester and Langridge, 2010), and agricultural production will need 50% more water. Making matters worse, freshwater is predicted to decrease by 50%, and approximately 5 billion people will live in water-scarce regions (Gupta et al., 2020). Water-use efficiency is defined as the grain yield or biomass production per unit of water used, and its improvement is considered one of the key traits for the adaptation to climate change (Hatfield and Dold, 2019). Therefore, it is premised that using soil water efficiently, while sustaining plant growth, is essential for water-saving agriculture. Hence, evaluating how efficiently water is used for plant growth and breeding crops that use less water has become imperative. Soybean (*Glycine max L*. Merr.), the most important leguminous crop, is cultivated globally for human food, animal feed, and biofuel (Valliyodan et al., 2017). Annual soybean production is largely challenged by several edaphic stresses, of which water deficiency is one of the major yield-limiting factors that can lead up to 40% of yield loss (Specht et al., 1999.). However, most of the breeding programs so far, including those for soybean, only focused on the above-ground parts of the plant and little research was conducted aiming at the root (Koevoets et al., 2016).

The root system plays a central role in water and nutrient uptake and is the first organ that senses and responds to soil conditions, e.g., water shortage (Chen et al., 2020). Recently, root system architecture (RSA) has been targeted in breeding and selection approaches and is proposed to be critically important for the second green revolution (Lynch, 2007; Villordon et al., 2014; Lynch, 2022). RSA refers to the roots’ shape, distribution, and branching pattern (Osmont et al., 2007; Prince et al., 2019), which are determined by root-related genetic background and its interaction with the environment (Valliyodan et al., 2017). In order for breeding programs to be successful, an in-depth understanding of natural variation and the underlying genetic basis is imperative (Iyer-Pascuzzi et al., 2010; Clark et al., 2013; Ye et al., 2018), justifying the increasing interest in evaluating the genetic variation in the RSA of crops (de Dorlodot et al., 2007; Kuijken et al., 2015; Chen et al., 2020). To date, studies have been conducted on the root systems of wheat (Chen et al., 2020), rice (Phung et al., 2016; Kadam et al., 2017; Guimarães et al., 2020), maize (Li et al., 2015), and soybean (Falk et al., 2020; Kim et al., 2021). However, most of these studies were carried out in controlled environments, with the exception of a study of Dhanapal et al. (2021), which investigated only the topsoil part of the root system. Phenotyping of roots under controlled conditions, including laboratories or greenhouses, can be done with a large number of genotypes at a reasonable cost. However, the growth conditions are space-limited and less effective in reflecting the actual performance of the roots (Ye et al., 2018; Takahashi and Pradal, 2021), which is highly dynamic and strongly influenced not only by water, nutrients, and soil temperature but also by the plant–soil interactions (Poorter et al., 2016). Thus, extensive and large-scale phenotyping of roots in the field is required to provide the basic data for selecting and breeding crops for mitigating consequences of climate change.

Until now, high throughput field root phenotyping remained a major challenge as soils are heterogeneous and opaque, and the plant–soil interactions are highly dynamic (Burridge et al., 2020). For that reason, numerous efforts have been made to improve the measurement and visualization of root systems. “Shovelomics,” the use of simple and robust excavation combined with field root visual scoring methods, has been widely used to quantify important root traits, including root length, root surface, root volume, taproot, and lateral root branching density in soybean (Fenta et al., 2014; Prince et al., 2019), crown root number and angle in maize (Trachsel et al., 2011), and basal root whorl number, basal root branching, and root growth angle in cowpea and common beans (Burridge et al., 2016). Recently, an estimation method for root depth for maize and common bean using soil coring was also reported (Burridge et al., 2020). Although the number of reports related to RSA is rapidly increasing, the root system remains poorly understood compared to the above-ground parts of the plants because of the constraints and difficulty in directly phenotyping the root system. This is a major bottleneck in the large-scale utilization of root traits to improve crop performance under both optimal and stressed environments (Koevoets et al., 2016; Falk et al., 2020). What is still missing to date is the establishment of a high-throughput phenotyping platform that is less time-consuming and labor-intensive and can be achieved with minimum cost. For this purpose, growing crops in special field conditions such as sandy soils has great potential as the specific soil environment allows not only robust root growth but also fast and convenient root phenotyping without a large loss of root.

The aim of this study was to investigate root growth in response to soil water as an indicator of water-use efficiency in soybean plants grown in an open field with a high sand content. For this purpose, we established a field irrigating system and cultivated 200 soybean accessions under non-irrigated or irrigated conditions. Subsequently, the genetic variation and effect of irrigation on root traits were evaluated, and the key root traits contributing to water use efficiency were identified. Finally, we applied several methods to select genotypes showing a large improvement of root traits under irrigated conditions and genotypes that exhibited both high performance and high stability of root growth across environments.

## 2 Materials and methods

### 2.1 Plant materials and field trials

**Figure 1:**
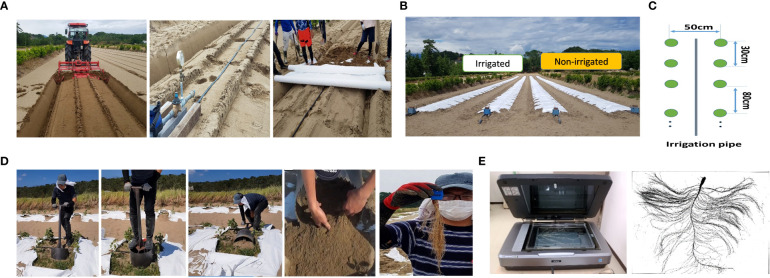
Field irrigation system and evaluation of root system architecture (RSA). A drip irrigation system was installed in the field of the Arid Land Research Center (ALRC), and the system was covered by mulching to avoid rain precipitation **(A)**; field-experiment layout **(B)**; planting density and location of irrigation pipe in the field **(C)**; steps for collecting roots system from the field **(D)**. The root system was evaluated using the WinRhizo system **(E)**.

In this study, we used a diverse soybean panel consisting of 200 accessions (**Supplementary Table 1**), of which 192 were from the Japanese mini-core (JMC) and world mini-core (WMC) collections provided by the National Agricultural and Food Research Organization (NARO) Genebank. The core collections were selected from 1603 soybean accessions based on morpho-agronomic trait variation, population structure, and geographic origin and were considered to retain 100% of the gene diversity (Kaga et al., 2012). Field trials were conducted at the Arid Land Research Center (ALRC), Tottori University, Japan (35°32’N, 134°12’E, 14 m above the sea level) in 2017, 2019, and 2020. The field is adjacent to the Tottori Sand Dune, and the soil comprises 96% sand, 1% silt, and 3% clay (Kimura et al., 2004). This texture enables good growth and makes destructive root sampling easy with minimum errors and reduced labor costs. Before the main experiments, a small soybean cultivation trial was performed in 2016, and from 2017 to 2020, the field was solely used for soybean cultivation. Each year’s experiment was laid out using a completely randomized design with two irrigation treatments: irrigated and non-irrigated conditions. Before sowing, the experimental ridges were covered with white mulch sheets (Tyvek, Dupond, USA) to prevent rainwater infiltration (**Figure 1**), as described by Toda et al. (2022). Uniform seeds were sterilized and sown at a 5-cm depth, and six plants were maintained in a single row plot (30 cm spacing; two plants/position) for each accession and treatment. Three of them were carefully selected for root phenotyping. Fertilizer (1.34, 1.18, 4.04, 1.66, and 2.24 g m^-2^ of N, P, K, Ca, and Mn, respectively) was applied once 2 weeks before sowing. Details of fertilization, including fertilizer types and ingredients, are provided in **Supplementary Table 2**.

A drip irrigation system was installed in the field by laying pipes (wall thickness:0.25 mm; inside diameter: 16 mm; dripper spacing: 10 cm; dripper flow: 1.1 L/h) in the middle of the growing bed under mulch sheets (**Figure 1**). In the irrigated plots, plants were watered daily after emergence (2 weeks after sowing) for 5 h (7:00–9:00, 12:00–14:00, 16:00–17:00), while no irrigation was performed in the non-irrigated plots until the day of root phenotyping. During the field experiment, soil moisture content across the field was recorded at a 30–40-cm depth from the soil surface using a hand-held soil moisture tester (TDR-341F, Fujiwara Seisakusho, Japan).The on-site rainfall data were also recorded. The detailed timeline of the field experiment, including the dates of sowing and root phenotyping in each year, is given in **Supplementary Table 3**.

### 2.2 Phenotyping and measurement of root traits

We used a self-designed tool that consists of a cylindrical blade (30 cm in diameter, 40 cm in height) with a handle (**Figure 1D**) to excavate the roots in the field. Using this tool, we were able to collect almost intact root systems of soybean (up to 40 cm depth from the field surface). After collection, the roots were quickly rinsed with water, transferred into zipped bags filled with 50 mL ethanol (50%) for storage, and finally scanned using the Expression 12000XL system (Epson, Japan). The 2D root images were loaded to the WinRHIZO Pro software (Regent Instruments Inc., Canada) to measure the following traits: total root length (TRL), total root surface area (Surface), average root diameter (Avd), total root volume (Vol), the total number of tips (Tips), length of thin root with diameter ≤ 0.4 mm (ThinRL), length of medium diameter root class from 0.4–1 mm (MidRL), and length of thick roots with diameter > 1 mm (ThickRL). We then calculated the proportion of the three TRL root classes, i.e., ThinRL_rate, MidRL_rate, and ThickRL_rate. Three first-order laterals were carefully selected from the whole root and individually scanned to calculate the number of tips emerging from each primary lateral per unit of length (tip/cm) to measure the secondary lateral root density (SLRD). Root dry weight (RDW) was manually measured after drying the roots at 80°C for 72 h. In addition to root traits, we investigated the shoot growth of soybean in response to field irrigation conditions by measuring two biomass-related traits, including shoot fresh weight (SFW) and plant height (PH). The detailed list of measured traits is given in **Supplementary Table 4**.

### 2.3 Statistical analysis

#### 2.3.1 Analysis of variance (ANOVA) and Shannon–Weaver diversity index

To estimate the significance of irrigation treatments and variation sources in each year, two-way ANOVA was employed to estimate the variation among the genotypes (G), the two treatments (T), and the interaction between genotypes and treatments (GxT). In addition, three-way ANOVA was also performed to evaluate the effects of genotype (G), treatment (T), and year (Y) across the three years of field experimentation.

We used the Shannon Weaver diversity index calculated based on estimated values obtained from the best linear unbiased prediction (BLUP) to compare the diversity of studied phenotypes within the whole panel and between the JMC and WMC collections. The “lme4” package in R (Bates et al., 2015) was used to calculate BLUP values based on a mixed model according to the following formula (Merk et al., 2012):


$$Y_{ik}=\text{μ}+G_{i}+Y_{k}+{GY}_{ik}+{\text{ϵ}}_{ik},$$

where Y_ik_ is the trait studied, μ is the overall mean, G_i_ is the i^th^ genotypic effect, Y_k_ is the effect of the k^th^ year, GY_ik_ is the interaction of genotype × year, and ϵ_ik_ is the residual error.

The BLUP values were used to classify the genotypes into three categories based on the overall mean and standard deviation: (i) genotypes with low trait values (BLUP ≤ µ-SD); (ii) genotypes with average trait values (µ-SD< BLUP ≤ µ+SD); and (iii) genotypes with high trait values (BLUP > µ-SD). The frequency of genotypes in each category was used to calculate the Shannon–Weaver diversity index (*H’*) according to Kumar et al. (2012) using the following formula:


$$H’=\sum_{i=1}^{n}\text{Pi logPi}$$

where Pi is the frequency of genotypes grouped in each category and n is the number of categories for a given trait (n = 3 in this study).

#### 2.3.2 Correlation and principal component analysis (PCA)

Pearson correlations were also calculated among root and shoot traits recorded in the two irrigation treatments using BLUP values. PCA using the prcomp function in R (version 4.0.5) was conducted on scaled BLUP values to understand the inter-relations among studied traits in the field.

#### 2.3.3 Screening for genotypes with large root improvement under irrigation

In order to identify genotypes with improved root architectural traits under irrigated treatment, we calculated the increment (I) index using the following formula:


$$\text{I}-\text{index }={\text{ Y}}_{\text{irrigated}}/{\text{Y}}_{\text{non-irrigated,}}$$

where Y_irrigated_ is the phenotype value of the irrigated test genotype, and Y_non-irrigated_ is the phenotype value of the non-irrigated test phenotype.

We also evaluated the increment in root traits and root performance values. For this, we considered calculating the stress tolerance index suggested by Fernandez (1992) and proposed the relative increment (RI) index, calculated using the following formula:


$$\text{RI}-\text{index}=\left({\text{Y}}_{\text{irrigated}}*{\text{Y}}_{\text{non-irrigated}}\right)/{\left({\text{X}}_{\text{non-irrigated}}\right)}^{2}$$

where Y_irrigated_ is the phenotype value of the test genotype under irrigated conditions, Y_non-irrigated_ is the phenotype value of test phenotype under non-irrigated conditions, and X_non-irrigated_ is the mean phenotype of test genotypes under non-irrigated conditions. The I-index and RI-index values calculated using BLUP data from 16 traits were then used for PCA in R software.

#### 2.3.4 Selection of genotypes based on multi-trait stability index (MTSI)

The analysis was conducted based on the singular value decomposition (SVD) of the matrix of BLUPs for the genotype-environment interaction effects (GEIs) obtained from a mixed linear model (LMM) to quantify the genotypic stability. This allows the selection of genotypes based on either only stability by quantifying the weighted average of absolute scores (WAASB values) from the SVD or estimating the WAASBY index, which allows the weighting between the mean performance (Y) and stability (WAASB). In this study, we chose to use simultaneous selection for both performance and stability (WAASBY), considering the ratio of 60% and 40% for mean performance and stability, respectively, prioritizing mean performance over stability (Olivoto et al. (2019). The WAASBY index was then used to calculate the MTSI index using 16 root and shoot traits, according to the following formula (Olivoto et al. (2019):


$$MTSI_{i}=\sum_{j=1}^{f}{\left[{\left(F_{ij}-F_{j}\right)}^{2}\right]}^{0.5}$$

where the MTSI is the multi-trait stability index for the ith genotype, Fij is the jth score of the ith genotype, and Fj is the jth score of the ideotype. The genotype observed with the lowest MTSI is closer to the ideotype, representing the high mean performance and stability for all analyzed variables.

The analyses were performed following the detailed instructions of Olivoto and Lúcio (2020) using the “metan” package in R software. The top 10% of genotypes with the lowest MTSI scores were selected and are highlighted in red in the MTSI plot.

## 3 Results

### 3.1 ANOVA of each year and across experimental years

The two-way ANOVA results revealed significant effects of G, T, and GxT for most of the root and shoot traits (**Supplementary Table 5**) in each year. The effects of T and G accounted for most of the variation in each year. Variations in the effects of T were observed over the years, as indicated by the F-values. The effects of irrigation (T) were highest in 2019, followed by 2017 and 2020.

Combined ANOVA for the three experimental years revealed the presence of highly significant variation for all the measured traits (**Supplementary Table 6**). The mean sum of squares for most traits showed significance for G, T, and Y. Overall, T and Y were the largest sources of the total variability (G+T+Y). The percent variation contributed to total variability by T was maximum at Surface (69.5%), followed by TRL (61.2%), ThinRL (61.2%), and Tips (55.5%), whereas Y shared about 90% for PH, followed by SLRD (85.8%), ThickRL_rate (83.8%), Avd (80.8%), and SFW (74.4%) of the total variation.

### 3.2 Responses of root and shoot traits to irrigation treatments

**Figure 2:**
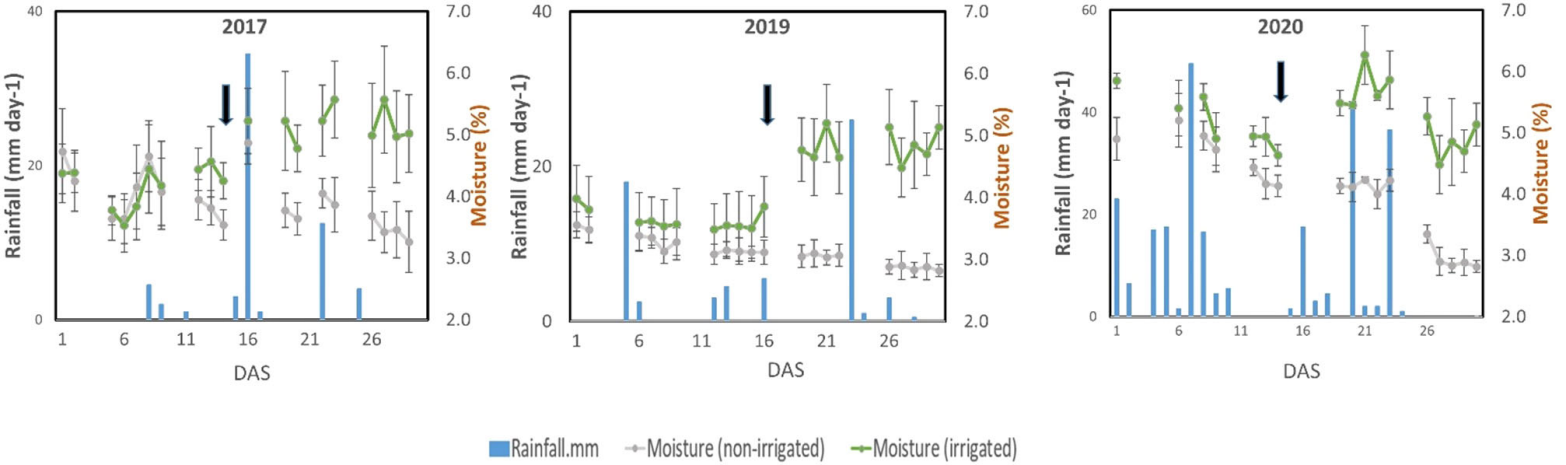
Field weather data (moisture content and rainfall) recorded during the experimental periods. Black arrows indicate the start of the irrigation treatment. Bars in the moisture content data points represent the standard deviation of means calculated usingf moisture content data measured in multiple positions in the field.

**Figure 3:**
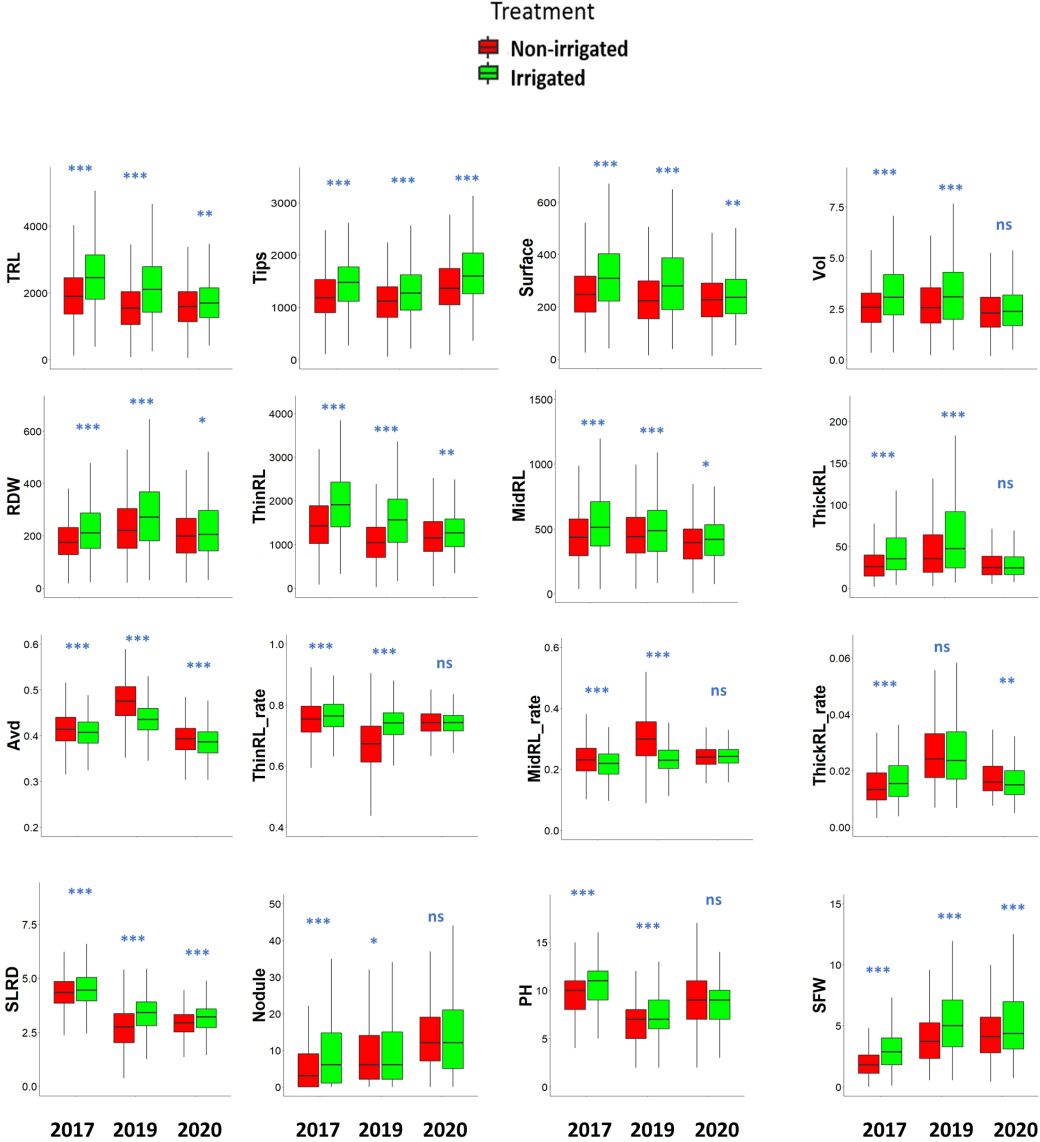
Root and shoot traits in response to irrigation treatments in a 200-genotype panel across 3 years. The data of each trait are described using box plots. Significant differences between non-irrigated and irrigated fields were analyzed for each trait using a t-test: ***p< 0.001; **p< 0.01; *p< 0.05; ns, not significant. TRL, total root length (cm); Tips: total number of root tips; Surface: total root surface area (cm^2^); Vol: Total root volume (cm^3^); RDW: root dry weight (mg); ThinRL: length of thin root with diameter ≤ 0.4 mm, MidRL: length of medium diameter root class from 0.4–1 mm, ThickRL: length of thick roots with diameter > 1 mm; ThinRL_rate: proportion of ThinRL in TRL; MidRL_rate: proportion of MidRL in TRL; ThickRL_rate: Proportion of ThickRL in TRL; Avd, average root diameter (mm); SLRD, secondary lateral root density (tip/cm); Nodule: nodule number per plant; PH, plant height (cm); SFW, shoot fresh weight (g).

Sandy soils cannot retain water, and without irrigation, the soil moisture content gradually decreased in the sandy field of ALRC. However, irrigation quickly restored the moisture content in the irrigated field (**Figure 2**). At root sampling, the moisture content was about 3% in the non-irrigated field but was maintained at 5% in the irrigated field. Responses to treatments were consistent among studied traits since most showed a significant increase under irrigated conditions, except for Avd, which showed a significant decrease (**Figure 3**). When considering the effects of irrigation on root traits, significantly high levels (*p<* 0.001) were observed for Avd, Tips, and SLRD each year. Most of the root traits showed significant differences between the two treatments each year, except for ThickRL_rate in 2019 and ThickRL, ThickRL_rate, ThinRL_rate, and nodule number in 2020. In addition, irrigation significantly improved plant biomass-related traits, including RDW and SFW. SFW was more sensitive to water content in the soil as an increase with a high significance level was observed each year, compared with PH, which only showed an increase in 2017 and 2019 under irrigation conditions.

### 3.3 Diversity of root traits in the panel

Irrigation improved most root traits each year; however, the average value of each trait occasionally varied over the years, indicating that the relative growth of the plants is affected by environmental conditions (**Figure 3**). As we noticed yearly differences in the trait values, BLUP value of each genotype was calculated using original data from the 3 years. The BLUP data showed a high correlation with data observed across the 3 years (*r^2 =^* 0.67 in 2017, *r^2 =^* 0.76 in 2019, *r^2 =^* 0.58 in 2020). The BLUP data also showed large variations among genotypes (**Table 1**; **Supplementary Figure 2**). Among the 16 shoot and root traits studied, 12 traits had a coefficient of variation (CV) value > 20% among genotypes. SFW and root traits such as dry weight (RDW), Vol, and ThickRL had larger variations (CV > 30%). ThickRL showed the highest variation among the genotypes, with a CV of 49.27% and 50.85% in irrigated and non-irrigated conditions, respectively. The range for ThickRL was 14.92–123.14 cm in irrigated conditions and 10.05–122.83 cm in non-irrigated conditions. In contrast, both treatments showed relatively low variation for Avd, thin root length (ThinRL), and SLRD with CV values< 10%.

**Table 1:** Variation and Shannon–Weaver diversity index (*H*’) in root and shoot traits among the whole panel and the Japanese and world mini-core collections, calculated using the best linear unbiased prediction(BLUP) data.

Trait	Treatment	All					Japanese mini-core			World mini-core		
		Min	Max	Range (%)	CV (%)	*H*’	Min	Max	*H’*	Min	Max	*H’*
**TRL**	irrigated	967.15	3424.64	354.10	25.75	*0.89*	1093.73	3424.64	*0.71*	967.15	3124.93	*0.81*
	*non-irrigated*	691.48	2843.25	411.19	25.87	*0.86*	1065.91	2843.25	*0.69*	691.48	2592.61	*0.79*
**Tips**	irrigated	774.76	2189.55	282.61	20.06	*0.88*	969.43	2176.95	*0.70*	774.76	2189.55	*0.83*
	*non-irrigated*	511.66	1906.67	372.64	22.06	*0.87*	778.98	1906.66	*0.70*	511.66	1870.04	*0.72*
**Surface**	irrigated	124.35	503.16	404.64	28.59	*0.88*	144.99	503.16	*0.72*	124.35	448.59	*0.79*
	*non-irrigated*	88.47	428.27	484.06	28.54	*0.88*	136.62	428.27	*0.71*	88.47	364.72	*0.78*
**Vol**	irrigated	1.26	5.75	454.72	32.21	*0.85*	1.49	5.75	*0.70*	1.26	4.98	*0.74*
	*non-irrigated*	0.90	5.44	600.87	32.06	*0.84*	1.37	5.44	*0.70*	0.90	3.97	*0.73*
**RDW**	irrigated	79.73	425.06	533.14	32.29	*0.85*	101.02	425.06	*0.68*	79.73	370.86	*0.76*
	*non-irrigated*	61.51	396.03	643.85	33.34	*0.87*	87.19	396.03	*0.71*	61.51	357.29	*0.76*
**ThinRL**	irrigated	738.81	2562.42	346.83	25.24	*0.88*	810.44	2562.42	*0.68*	738.81	2286.78	*0.83*
	*non-irrigated*	508.96	1997.30	392.43	25.58	*0.87*	837.48	1997.30	*0.69*	508.96	1972.38	*0.80*
**MidRL**	irrigated	196.82	829.12	421.26	28.54	*0.86*	272.78	829.12	*0.72*	196.82	730.70	*0.73*
	*non-irrigated*	145.48	761.54	523.47	28.62	*0.80*	215.94	761.54	*0.68*	145.48	674.49	*0.74*
**ThickRL**	irrigated	14.92	123.14	825.55	49.27	*0.85*	16.19	123.14	*0.74*	14.92	99.08	*0.76*
	*non-irrigated*	10.05	122.83	1222.61	50.85	*0.80*	15.58	122.82	*0.69*	10.05	71.94	*0.70*
**Avd**	irrigated	0.36	0.47	131.03	5.68	*0.85*	0.36	0.47	*0.77*	0.36	0.45	*0.74*
	*non-irrigated*	0.37	0.49	132.25	5.21	*0.85*	0.37	0.49	*0.74*	0.38	0.49	*0.78*
**ThinRL_rate**	irrigated	0.65	0.83	127.94	4.19	*0.87*	0.68	0.82	*0.76*	0.65	0.83	*0.79*
	*non-irrigated*	0.61	0.81	131.66	4.74	*0.86*	0.65	0.80	*0.77*	0.61	0.08	*0.77*
**MidRL_rate**	irrigated	0.16	0.33	208.66	12.12	0.84	0.17	0.29	0.70	0.16	0.33	0.81
	*non-irrigated*	0.18	0.38	210.30	12.65	0.88	0.18	0.32	0.75	0.18	0.38	0.81
**ThickRL_rate**	irrigated	0.01	0.04	341.05	29.03	0.80	0.01	0.04	0.73	0.01	0.04	0.73
	*non-irrigated*	0.01	0.05	454.86	31.24	0.73	0.01	0.05	0.74	0.01	0.03	0.65
**SLRD**	irrigated	3.05	4.25	139.25	6.26	0.86	3.05	4.15	0.72	3.06	4.25	0.82
	*non-irrigated*	2.57	4.14	161.05	7.55	0.84	2.57	4.14	0.72	2.57	3.91	0.80
**Nodule**	irrigated	5.53	38.33	693.14	38.52	0.78	6.86	38.32	0.70	5.52	24.08	0.74
	*non-irrigated*	3.69	29.87	809.23	47.57	0.74	4.26	29.87	0.73	3.69	22.87	0.69
**PH**	irrigated	4.82	14.92	309.83	18.56	0.83	6.63	14.92	0.73	4.81	12.64	0.71
	*non-irrigated*	4.83	14.98	310.38	19.03	0.82	6.23	14.98	0.68	4.82	12.56	0.73
**SFW**	irrigated	1.16	9.66	834.50	33.66	0.88	1.15	9.66	0.71	1.15	6.90	0.76
	*non-irrigated*	1.01	8.41	830.01	36.90	0.87	1.04	5.41	0.69	1.01	5.38	0.79

TRL, total root length (cm); Tips, total number of root tips; Surface, total root surface area (cm^2^); Vol, Total root volume (cm^3^); RDW, root dry weight (mg); ThinRL, length of thin root with diameter ≤ 0.4 mm; MidRL, length of medium diameter root class from 0.4–1 mm; ThickRL, length of thick roots with diameter > 1 mm; ThinRL_rate, proportion of ThinRL in TRL; MidRL_rate, proportion of MidRL in TRL; ThickRL_rate, Proportion of ThickRL in TRL; Avd, average root diameter (mm); SLRD, secondary lateral root density (tip/cm); Nodule, nodule number per plant; PH, plant height (cm); SFW, shoot fresh weight (g).

The *H’* calculated for the whole panel and the two mini-collections (JMC and WMC) are shown in **Table 1**. Most studied traits had high diversity (*H’* > 0.8) across the panel under both irrigated and non-irrigated conditions. No noticeable differences in the *H’* values were found between the irrigation treatments. Higher *H’* values were found in the WMC genotypes for most traits. Notably, the variation in *H’* values between the mini-core collections was largely seen in TRL, Tips, and ThinRL. For instance, in the case of TRL, the *H’* values were 0.81 and 0.79 for TRL in WMC compared with 0.70 and 0.69 for JMC under irrigated and non-irrigated conditions, respectively. The larger diversity in TRL was also reflected in the variation in the ranges of TRL in the two mini-core collections, especially in non-irrigated treatments. The TRL ranged from 691.48 to 2592.61 cm (equivalent to 374.8%) in WMC, whereas a smaller range of 267% (from 1065.91–2843.25 cm) was observed in JMC. In addition to observing variations in the *H’* values between the two mini-core collections, we investigated the variations in TRL and SFW among different groups based on the origin of accessions (**Supplementary Figure 2**). The results showed that accessions from Japan and Korea had relatively higher TRL and SFW values, followed by accessions from China and Taiwan. The accessions from Southeast Asia and South Asia (India, Nepal, and Pakistan) had the lowest TRL and SFW values (**Supplementary Figure 2**).

### 3.4 Correlation between root and shoot traits and PCA

**Figure 4:**
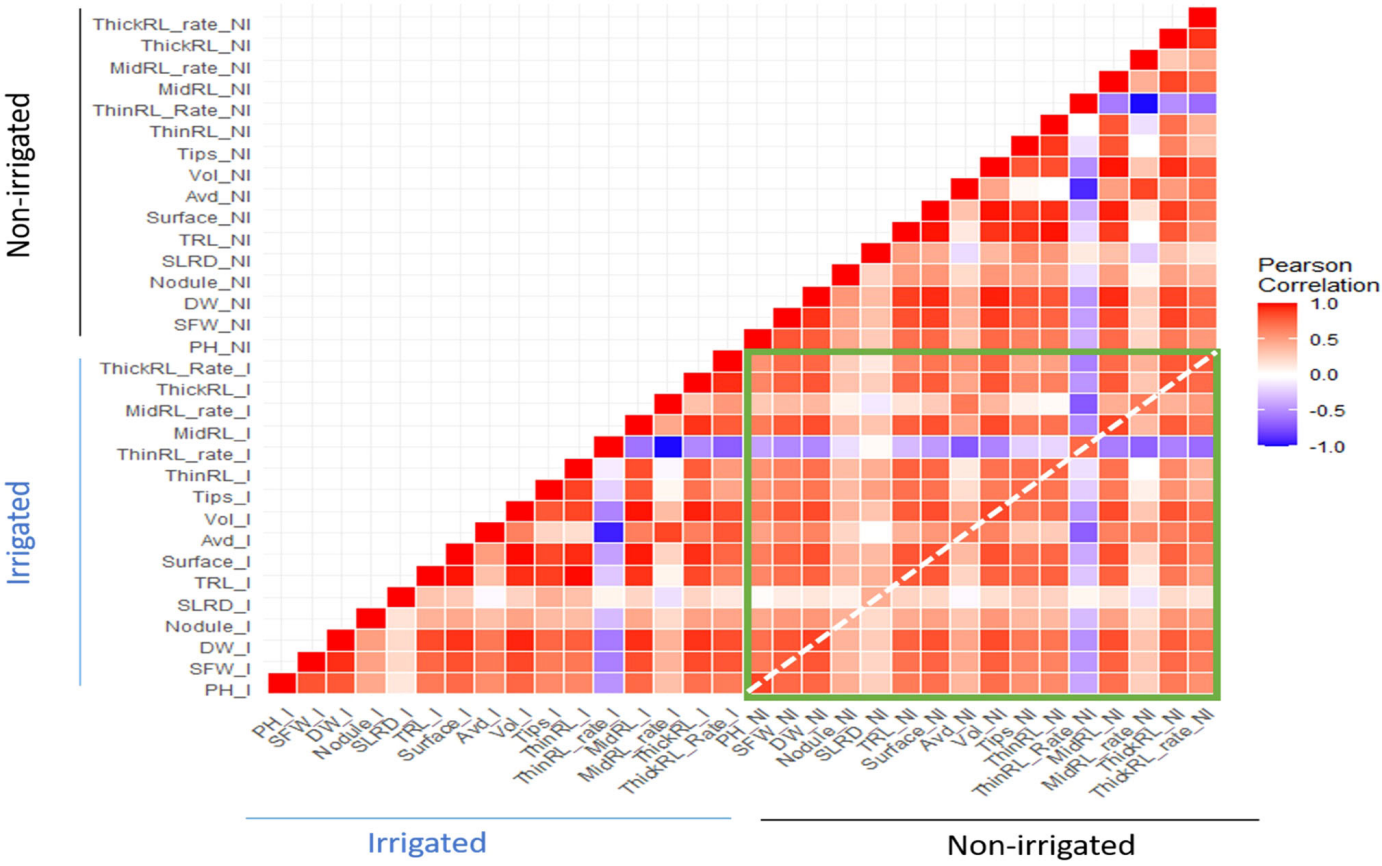
Heatmap showing the correlations between root and shoot traits within and between two irrigation treatments. The green rectangle shows the correlation of traits in non-irrigated conditions with traits in irrigated conditions; the white-dotted line highlights the correlation between the traits in the two irrigation treatments.

**Figure 5:**
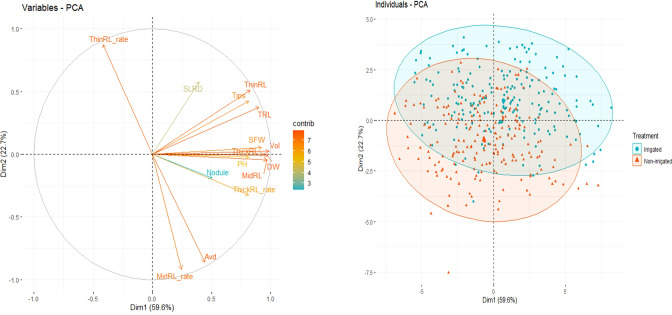
Principal component (PC) analysis bi-plot showing the two first PCs using best linear unbiased prediction (BLUP) values of 16 root and shoot traits (left) and grouping of individual genotypes by irrigation treatment (right).

Pearson correlations among shoot and root traits were calculated, and significant correlations (*p*<0.01) were observed in most of the pairs (**Figure 4** and **Supplementary Figures 3, 4**). High correlations were found among TRL, Surface, and ThinRL, and between Vol and MidRL (*r* > 0.9) under both irrigated and non-irrigated conditions (**Figure 5**, **Supplementary Figures 3, 4**). Among the two studied shoot traits, SFW showed a higher correlation with root traits, including RDW (*r* > 0.8) and ThickRL (r > 0.7), in both treatments. While positive correlations were found in most of the pairs of studied traits, ThinRL_rate was negatively correlated with most of the traits, especially with Avd. No noticeable difference in correlations among studied traits was found between the two irrigation treatments, except for the correlations between Avd and RDW and between Avd and Vol (**Figure 5**). Under the irrigated conditions, Avd showed a higher correlation with RDW and Vol with r values of 0.68 and 0.63 compared with 0.47 and 0.46 under non-irrigated conditions, respectively. Low correlations were found for Nodule and SLRD with the other studied traits, suggesting that these traits might vary among different irrigation conditions.

PCA using BLUP values of the 16 root and shoot traits revealed that the first two principal components (PCs) explained approximately 83.2% of the total variation among all accessions in the experiment (**Figure 5**). The first PC, explaining 59.6% of the total variation, was mainly contributed by traits related to the size of the root system, i.e., Vol, Surface, MidRL, ThickRL, RDW, TRL, and SFW (**Supplementary Figure 1**). The most important traits in PC2, contributing to 22.7% of the total variation, were Avd, ThinRL_rate, and MidRL_rate (**Supplementary Figure 1**).

### 3.5 Identification of genotypes with improved root traits under irrigated conditions

Both the I- and RI-indices showed high variation between the genotypes, but there was no consistency between these indices (**Supplementary Figure 5**). A higher variation was observed in the RI-index than in the I-index for all the studied traits (**Supplementary Figure 5**).

**Figure 6:**
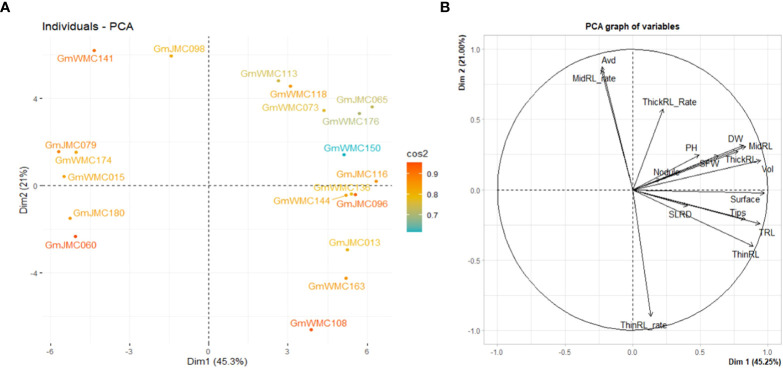
Bi-plot showing the top 20 genotypes with the highest contribution **(A)** and relation among variables for the first two components **(B)** obtained in principal component (PC) analysis using the increment- index calculated for 16 shoot and root traits. The color scale indicates the contribution of genotypes to the first two PCs by cos^2^ values.

PCA using I-index revealed that the first two PCs explained 66.3% of the total variation. Thus, it was plausible to select genotypes using the first two PCs. We then evaluated the top 20 genotypes that showed the highest contribution to the total variation in the I-index (**Figure 6**). Based on PC1, genotypes with highly positive component values in PC1, including GmJMC065, GmJMC116, GmJMC096, GmJMC013, and GmWMC163, exhibited relatively greater improvements in Vol, TRL, and RDW. However, in PC2, GmJMC013 and GmWMC163 had relatively higher increments in ThinRL_rate and smaller increments in Avd compared with GmJMC065 and GmJMC116, as indicated by their high negative PC scores. This suggested that the root traits of these genotypes largely improved under irrigation. Among them, GmJMC065 and GmJMC116 were more sensitive to water content levels. In contrast, certain genotypes, including GmJMC079, GmWMC174, GmWMC015, GmJMC180, and GmJMC060, showed relatively low I-index PC1 scores, indicating that water supply may not be a limiting factor for their root development.

**Figure 7:**
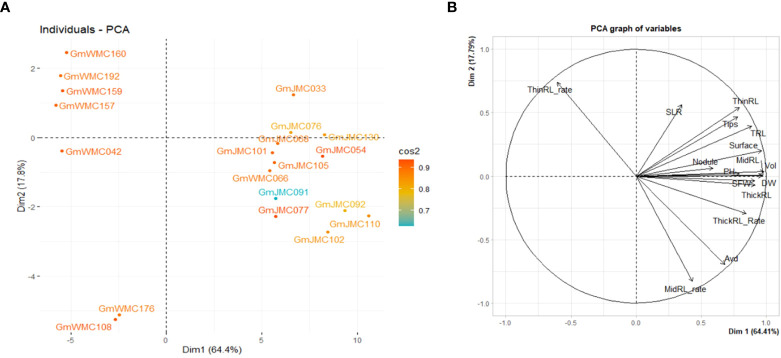
Bi-plot showing the top 20 genotypes with the highest contribution **(A)** and relation among variables for the first two components **(B)** obtained in principal component (PC) analysis using the relative increment index calculated for 16 shoot and root traits. The color scale indicates the contribution of genotypes to the first two PCs by cos^2^ values.

The I-index, unlike the RI-index, did not consider the large or small trait values of each genotype in relation to other genotypes in the panel. Using the RI-index, we concluded that the first two PCs accounted for 82.1% of the total variation in the panel. Among the top 20 genotypes with the highest contribution to the first two PCs, most of the genotypes with high positive PC1 scores were in the JMC collection (**Figure 7**). Genotypes with the highest PC1 scores were GmJMC110, GmJMC092, GmJMC102, GmJMC130, and GmJMC054. The high positive PC1 scores observed in these genotypes indicated that they exhibited relatively high RI-index values in most of the root traits in PC1, including Vol, RDW, and TRL. In contrast, genotypes with highly negative PC1 scores were from the WMC collection: GmWMC160, GmWMC192, GmWMC159, GmWMC157, and GmWMC042. These genotypes had small root systems and relatively low RI-index values in most of the important root traits in PC1, such as Vol, RDW, and TRL. GmWMC160 and GmWMC192 had high positive PC2 scores among these genotypes, suggesting they had a high RI-index value in ThinRL_rate and relatively small increments in Avd.

### 3.6 Identification of genotypes with high stability and performance across environments

**Figure 8:**
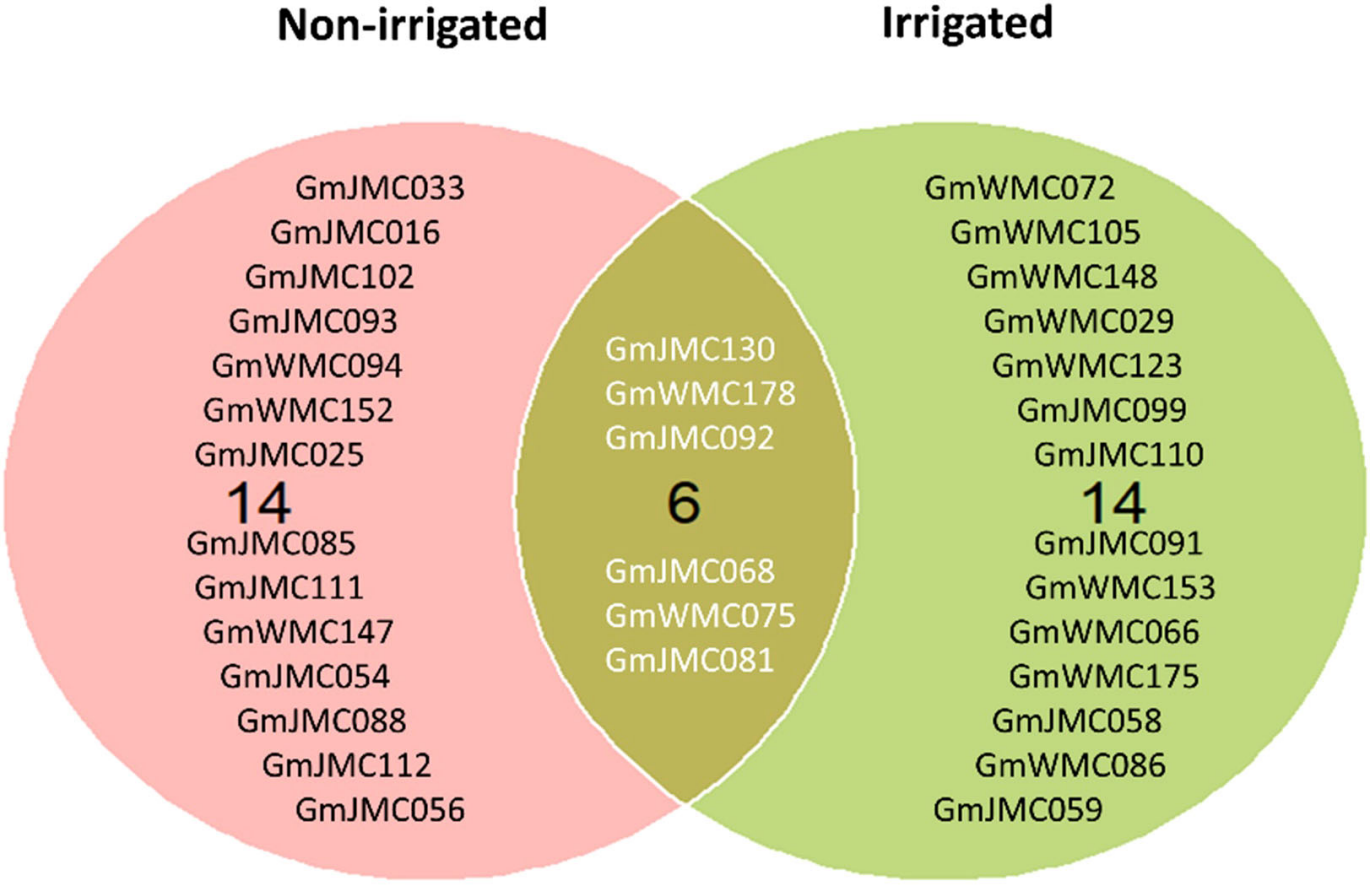
Selection of the top 10% of genotypes with high stability and high performance across years under irrigated and non-irrigated conditions using the multi-trait selection index of 16 root and shoot traits.

The selection of genotypes was performed using the MTSI index values calculated based on the 16 root and shoot traits. The calculated values of indices used for the simultaneous selection of genotypes are provided in **Supplementary Tables 7, 8**. Genotypes with lower MTSI socres, found to be closer to the ideotype, were selected (**Supplementary Table 9** and **Supplementary Figures 8, 9**). Among the top 10% of the best genotypes with high performance and stability of root growth under irrigation across years, 10 were from Japan, and 10 were from WMC, whereas under non-irrigated conditions, 15 genotypes were from Japan, and 5 genotypes from WMC. Interestingly, six genotypes, namely, GmJMC130, GmWMC178, GmJMC092, GmJMC068, GmWMC075, and GmJMC081, were selected under both conditions (**Figure 8**). Among these genotypes, GmJMC092 and GmJMC130 had relatively large improvements in root traits such as Vol, TRL, and RDW.

## 4 Discussion

### 4.1 Root growth in response to irrigation

Our study is the first to investigate the variation in root system traits among the JMC and WMC collections of soybean grown in field conditions. Specifically, this study provides information on root phenomes resulting from one of the biggest field experiments, which was repeated for 3 years with two irrigation treatments, allowing the evaluation of both performance and stability of root growth. Genotype and treatment had a significant effect on shoot biomass and root traits, although the effect of year was greater than the above effects for several traits(**Supplementary Tables 5, 6**). It is known that weather parameters such as temperature, solar radiation, and rainfall affect bothe shoot and root growth (Poorter et al., 2016). Thus, the high SFW in 2019 and 2020 can be linked to the higher temperature recorded in the field compared to that observed in 2017. In addition, the high rainfall recorded in 2020 probably had an influence on root growth under both irrigation treatments. Therefore, we also calculated BLUP values that took into account genotypic and year effects, as it was difficult to directly compare the effect with or without irrigation on plant growth across years.

Water acquisition by plants is considered to be strongly linked with the spatial distribution of water in soil (Lynch, 2013; Lynch et al., 2021). In this study, irrigation improved all traits related to root system size, except Avd, which was reduced (**Figure 1**). Under irrigation, plants tend to have smaller Avd, increased Tips, and increased SLRD, suggesting that these traits exhibited an early response to water content and irrigation conditions and, thus, can be considered indicators of root trait improvement due to field irrigation. Root diameter and tissue density, which were highly correlated with root length and root surface, are known to affect the interacting space between the root and soil (Comas et al., 2013) and the colonization by mycorrhizal fungi, assisting nutrient acquisition by the roots (Smith and Read, 2008). Specifically, roots with smaller diameter roots and a higher number of root tips will allow the root system to maximize the contact area with soil water and lower the apoplastic barrier of water entering the xylem, increasing root hydraulic conductivity (Solari et al., 2006; Hernández et al., 2010; Comas et al., 2013).

PCA using BLUP values of each trait showed that root traits related to the size of the plant or root system (e.g., Vol, RDW, and TRL) contributed to PC1. In contrast, Avd, ThinRL_rate, and MidRL_rate mainly contributed to PC2. The above traits were the most important and explained most of the total phenotypic variation between tested genotypes. These results are in agreement with those from previous studies (Kumar et al., 2012; Li et al., 2015; Reddy et al., 2020), suggesting that a few key traits can characterize the root system, and it is more economical to concentrate on one key trait, such as RDW, than on root length traits, which are time-consuming and labor-intensive (Kumar et al., 2012). However, from a breeding point of view, the selection criteria must be aligned with diverse needs and interests. Therefore, all root traits should be considered to have great potential for root improvement. For instance, while high water and nutrient uptake are associated with fine roots and a fibrous root system (Henry et al., 2011), having thick roots (roots with large diameters) enables deeper penetration during drought and in compacted soils (Clark et al., 2008; Lynch et al., 2014; Yamauchi et al., 2021).

### 4.2 Identification of genotypes with promising root system in response to irrigation

Natural variation in root traits has been exploited to improve the root system in soybean (Manavalan et al., 2009; Chen et al., 2022). The results of this study suggested large variations in several root traits, as shown by high CV values for ThickRL, TRL, RDW, and Vol (**Table 1**), indicating a plasticity of root phenotype to quickly respond to environmental change at a given place and time (Zhu et al., 2005; Burridge et al., 2016). Additionally, since soybean was grown in a sandy field, root elongation and initiation were less restricted by soil friction and compaction level, compared with those when grown in clay and loamy soils, thus allowing the maximum growth of length and length-related traits. Furthermore, we calculated the *H’*, which has been widely used to assess the diversity of root traits in various crops such as rice (Bajracharya et al., 2006), maize (Kumar et al., 2012; Li et al., 2015), wheat (Lin et al., 2019), and cowpea (Adu et al., 2019). Although most of the studied traits had high *H’* values (> 0.8) under both irrigation treatments, the differences in the *H’* values between the treatments were not significant for most traits, suggesting that our soybean panel is genetically diverse, resulting in high root plasticity in response to soil water content in the soil. Variations in root traits were also detected in soybean accessions from different origins. Soybean from the JMC collection had larger root systems compared with soybean from the WMC collection. In Japan, the breeding programs for soybean are mostly focused on seed quality and food quality treats, such as large seeds and high protein content (Kaga et al., 2012). Soybean originating from Japan and Korea showed a relatively large root system (**Supplementary Figure 2**), and a correlation between seed size and TRL (data not shown). This explains the uniqueness of the genetic resources of Japanese soybean (Abe et al., 2003; Li et al., 2020; Kajiya-Kanegae et al., 2021).

With the recent availability of large root phenomes, screening for the desired root phenotype within many genotypes should be easy, rapid, and inexpensive (Grzesiak et al., 2019). The plasticity in root traits can be a breeding target (Schneider and Lynch, 2020), which we aimed to investigate by evaluating the sensitivities of soybean root growth in response to irrigation. For this purpose, we followed two approaches to screen for promising genotypes. Under irrigation, the response of the root system was highly plastic, as seen by the high variation in the I- and RI-index values. This suggested variations in the sensitivities to irrigation among genotypes in the panel. Therefore, for root trait improvement by irrigation, we selected genotypes that showed high PC1 values in PCA using the I-index and RI-index of root traits. Among the top 20 genotypes showing the highest contribution to PC1 and PC2, genotypes with high I- or RI-index values (high PC1 scores) increased TRL under irrigation. However, the I-index is calculated as the ratio of trait values under irrigation over the respective values under non-irrigation and does not consider the magnitude of trait values. Therefore, high I-index plants included plants that showed both large and small trait values. In contrast, the RI-index included this information on each trait, and plants with small trait values were ranked as low RI-index plants. Therefore, the average TRL was much higher in the genotypes selected with the RI-index than in those selected with the I-index. Thus, we considered that the method based on the PCA analysis using the I-index and RI- indices was appropriate for selecting genotypes with promising root systems.

Selecting promising genotypes with high stability and growth across diverse environments is fundamental for adapting to climate change. Recently, the use of the MTSI suggested by Olivoto et al. (2019) has been widely used in germplasm evaluation and selection of various crops, including maize under different moisture regimes (Singamsetti et al., 2021), soybean under drought and saline stress (Zuffo et al., 2020), and bread wheat adapted to early sowing conditions (Farhad et al., 2022). We applied the MTSI to select genotypes with high stability and performance in multiple root and shoot traits over 3 years of experimentation under two irrigation treatments. Among the selected genotypes under non-irrigated conditions, GmJMC025 (cv. Enrei) is one of the major cultivars in Japan. In addition, some genotypes were also selected using the RI-index, which considered the performance of each trait. This suggested that using MTSI could provide promising results for germplasm evaluation and selection in soybean grown across environments. We focused on genotypes that showed high plasticity in root growth in response to irrigation. However, genotypes with negative PC1 values in the I-index would be interesting materials for root improvement. It is suggested that water might not be the limiting factor for root growth because these genotypes exhibited better or similar growth under non-irrigated conditions than under that in irrigated conditions. Drought tolerance is one of the important traits for water-saving agriculture; these genotypes may show high tolerance to drought stress and contribute toward this achievement.

## 5 Conclusions

Large-scale field root phenotyping was applied in our study to evaluate the effects of field irrigation and genetic variation in root traits in a 200-genotype panel of soybean across 3 years. The growth of soybean root and shoot was significantly affected by irrigation treatments. The responses of root traits to irrigation treatments were consistent across the years, as most of the studied traits showed an increase under irrigated conditions. Among them, root dry weight, total root length, and root volume were the key traits contributing to the variation among the whole panel. Moreover, significant differences between irrigation treatments were observed each year in root average diameter, total number of root tips, and secondary lateral root density, suggesting that these traits as indicators for the early response of root traits to irrigation conditions. We also found a high diversity in root traits across the whole panel. It is worth noting that higher diversity in root traits was recorded in genotypes from the world mini-core collection, which can be exploited to broaden the genetic base of Japanese soybean through selection and breeding programs. Given the high diversity in root traits, we attempted to select promising genotypes using different approaches. While the selection of genotypes based on the increment index highlighted accessions with high plasticity toward irrigation conditions and can be used for parental lines for breeding or the mapping of quantitative trait locus for root trait improvement, genotypes with high relative increment in root traits had high values of root traits as well as underwent great improvement in root traits with irrigation. Specifically, the selection based on the multi-trait selection index yielded some shared genotypes under irrigated and non-irrigated conditions. These genotypes showed high stability and showed high growth performance across the two irrigation treatments in the 3 years of experimentation. One of the limitations of our study is that the root sampling was only done at an early growth stage of soybean to optimize for the most intact roots. To overcome this, future studies can be extended to the latter growth stages with analysis of yield and yield-related traits to determine whether the high performance and high stability of root traits are also related to high and stable crop yield.

## Data availability statement

The original contributions presented in the study are included in the article/**Supplementary Material**. Further inquiries can be directed to the corresponding authors.

## Author contributions

KTB, TN, HY, YT, YO, MT, AK, YY, HTs, YI, MH, TF, HI, MM, HTa, and MN contributed to the field experiment in ALRC. KTB, HTa, and MN prepared the manuscript, and all authors advised on the preparation of the manuscript. All authors have reviewed and approved the final manuscript.
