# Supplementary material for: Effects of irrigation on root growth and development of soybean: A 3-year sandy field experiment
Source: Front Plant Sci. 2022 Dec 14;13:1047563. doi: 10.3389/fpls.2022.1047563 (PMC9795411; doi:10.3389/fpls.2022.1047563)
Supplement: Supplementary file 1 [file DataSheet_1.docx]

**SUPPLEMENTARY MATERIAL**

**Supplementary Table 1**. List of the 200 soybean genotypes used in the study

| **Genebank accession**  **name** | **Accession Name** | **Status** | | **Origin** |
| --- | --- | --- | --- | --- |
| GmWMC001 | FISKEBY V | Breeding line | | Sweden |
| GmJMC002 | WASE KURO DAIZU | Landrace | | Japan |
| GmJMC003 | NATSU KURAKAKE | Landrace | | Japan |
| GmJMC004 | KITAJIRO | Landrace | | Japan |
| GmJMC005 | WASEOUSODE (SHIKAOI ITOH) | Landrace | | Japan |
| GmWMC006 | KS 1034 | Landrace | | Malaysia |
| GmJMC007 | TOKACHI NAGAHA | Breeding line | | Japan |
| GmJMC008 | KANAGAWA WASE | Landrace | | Japan |
| GmJMC009 | SHIZUNAIDAIZU | Breeding line | | Japan |
| GmWMC010 | DA BAI MEI | | Landrace | China |
| GmWMC011 | SEITA | | Landrace | Rep.Korea |
| GmWMC012 | MANSHUU | | Landrace | China |
| GmJMC013 | CHIZUKA IBARAKI 1 | | Breeding line | Japan |
| GmWMC014 | KLS 203 | | Landrace | Rep.Korea |
| GmWMC015 | CHUUHOKU 2 | | Landrace | Rep.Korea |
| GmJMC016 | JUKKOKU | | Landrace | Japan |
| GmJMC017 | SAKYUU AO MAME | | Landrace | Japan |
| GmWMC018 | RIGAI SEITOU | | Landrace | China |
| GmWMC019 | CHOUSENSHU(CHA) | | Landrace | Korean Peninsula |
| GmWMC020 | POCHAL | | Landrace | Taiwan |
| GmJMC021 | OOYACHI 2 | | Breeding line | Japan |
| GmWMC022 | NEZUMI META | | Landrace | Korean Peninsula |
| GmJMC023 | KUROGOYOU | | Landrace | Japan |
| GmWMC024 | CHIENEUM KONG | | Landrace | Rep.Korea |
| GmJMC025 | ENREI | | Breeding line | Japan |
| GmJMC026 | ONI HADAKA | | Breeding line | Japan |
| GmWMC027 | KONGNAMUL KONG | | Landrace | Rep.Korea |
| GmJMC028 | KOITO | | Landrace | Japan |
| GmWMC029 | SHIROSOTA | | Landrace | Korean Peninsula |
| GmJMC030 | KURODAIZU(AO HIGUU CHUU) | | Landrace | Japan |
| GmJMC031 | SHIRO MITSU MAME | | Landrace | Japan |
| GmJMC032 | NATTOU KOTSUBU | | Breeding line | Japan |
| GmJMC033 | BANSEI HIKARIKURO | | Landrace | Japan |
| GmJMC034 | MISOMAME | | Landrace | Japan |
| GmWMC035 | PEKIN DAI OUTOU | | Landrace | China |
| GmWMC036 | MASSHOKUTOU(KOU 502) | | Landrace | China |
| GmJMC037 | YAKUMO MEAKA | | Landrace | Japan |
| GmWMC038 | ICHIGUUHOU | | Landrace | China |
| GmJMC039 | NATTOUMAME | | Landrace | Japan |
| GmJMC040 | KOIBUCHIMURA ZAIRAI | | Landrace | Japan |
| GmJMC041 | DATE CHA MAME | | Landrace | Japan |
| GmWMC042 | MASSHOKUTOU(KOU 503) | | Landrace | China |
| GmJMC043 | TAKIYA | | Landrace | Japan |
| GmJMC044 | SHAKKIN NASHI | | Landrace | Japan |
| GmWMC045 | OKJO | | Landrace | Rep.Korea |
| GmWMC046 | KE 32 | | Landrace | Philippines |
| GmJMC047 | AKITA ANI | | Landrace | Japan |
| GmWMC048 | HEAMNAM | | Landrace | Rep.Korea |
| GmJMC049 | HIKU ANDA | | Landrace | Japan |
| GmJMC050 | FUKUI SHIRO | | Landrace | Japan |
| GmJMC051 | KURODAIZU(GEIHOKU) | | - | Japan |
| GmJMC052 | KISAYA(NATSU) | | Landrace | Japan |
| GmJMC053 | ABURA MAME | | Landrace | Japan |
| GmJMC054 | ZAIRAI 51-6 | | Landrace | Japan |
| GmJMC055 | SAKURAMAME | | Landrace | Japan |
| GmJMC056 | TAMAHOMARE | | Breeding line | Japan |
| GmJMC057 | SHAKUJOU MAME | | Landrace | Japan |
| GmJMC058 | YAHAGI | | Landrace | Japan |
| GmJMC059 | SOKOSHIN | | Landrace | Japan |
| GmJMC060 | SHIMO HISAKATA DAIZU | | Landrace | Japan |
| GmJMC061 | KOMAME | | Landrace | Japan |
| GmJMC062 | AZEMAME | | Landrace | Japan |
| GmJMC063 | AOBAKO | | Landrace | Japan |
| GmJMC064 | MEGURO 1 | | Landrace | Japan |
| GmJMC065 | OOJIRO | | Landrace | Japan |
| GmWMC066 | HEUKDAELIP | | Landrace | Rep.Korea |
| GmJMC067 | HOUJAKU | | Landrace | Japan |
| GmJMC068 | ZAIRAI 51-2 | | Landrace | Japan |
| GmJMC069 | CHADAIZU | | Landrace | Japan |
| GmWMC070 | CHOYOUTOU | | Landrace | China |
| GmWMC071 | PK 73-54 | | Landrace | India |
| GmWMC072 | M 581 | | Landrace | India |
| GmWMC073 | URONKON | | Landrace | Korean Peninsula |
| GmWMC074 | THET LAT(3 MONTH) | | Landrace | Myanmar |
| GmWMC075 | CHEONGYE MYONGTAE | | Landrace | Rep.Korea |
| GmJMC076 | IHHON SANGOU | | Landrace | Japan |
| GmJMC077 | HITORIMUSUME | | Landrace | Japan |
| GmJMC078 | AKASAYA | | Landrace | Japan |
| GmJMC079 | KURUMIMAME | | Landrace | Japan |
| GmJMC080 | HIME DAIZU | | Landrace | Japan |
| GmJMC081 | AKUDEN SHIRAZU | | Landrace | Japan |
| GmJMC082 | AOAKIMAME | | Landrace | Japan |
| GmWMC083 | KEUMDU | | Landrace | Rep.Korea |
| GmWMC084 | PEKING | | Landrace | China |
| GmJMC085 | DAIZU | | Landrace | Japan |
| GmWMC086 | ANTO SHOUKOKUTOU | | Landrace | China |
| GmWMC087 | DAU TUONG XANH LO | | Landrace | Viet Nam |
| GmJMC088 | CHUU TEPPOU | | Landrace | Japan |
| GmWMC089 | BONGCHUNBAEKJAM | | Landrace | China |
| GmJMC090 | DADACHAMAME | | Landrace | Japan |
| GmJMC091 | KUROTOME | | Landrace | Japan |
| GmJMC092 | KUROHIRA | | Landrace | Japan |
| GmJMC093 | ZAI 52-12 | | Landrace | Japan |
| GmWMC094 | JEOKGAK | | Landrace | Rep.Korea |
| GmJMC095 | AKASAYA | | - | Japan |
| GmJMC096 | NAKAHATA ZAIRAI | | Landrace | Japan |
| GmJMC097 | IPPON SUZUNARI | | Landrace | Japan |
| GmJMC098 | AKA DAIZU | | Landrace | Japan |
| GmJMC099 | AMAGI ZAIRAI 90D | | Landrace | Japan |
| GmJMC100 | KUROMAME | | Landrace | Japan |
| GmJMC101 | COL/EHIME/1983/UTSUNOMIYA 22 | | Landrace | Japan |
| GmJMC102 | KURAKAKE | | Landrace | Japan |
| GmWMC103 | SENYOUTOU | | Landrace | China |
| GmJMC104 | DAIZU(SHIRO) | | Landrace | Japan |
| GmJMC105 | MAETSUE ZAIRAI 90B | | Landrace | Japan |
| GmJMC106 | HIMESHIRAZU | | Breeding line | Japan |
| GmWMC107 | HAKKA ZASHI | | Landrace | China |
| GmWMC108 | KARASUMAME | | Landrace | China |
| GmWMC109 | TOPUTOPPU | | Landrace | Thailand |
| GmJMC110 | COL/TANBA/1989/ODAGAKI 2 | | Landrace | Japan |
| GmJMC111 | AMAGI ZAIRAI 90A | | Landrace | Japan |
| GmJMC112 | FUKUYUTAKA | | Breeding line | Japan |
| GmWMC113 | BARITOU 3 A | | Landrace | Indonesia |
| GmJMC114 | COL/EHIME/1-2 | | - | Japan |
| GmWMC115 | WILLIAMS 82 | | Breeding line | USA |
| GmJMC116 | SHIRATAMA | | Landrace | Japan |
| GmJMC117 | AKISENGOKU | | Breeding line | Japan |
| GmWMC118 | OUDU | | Landrace | Rep.Korea |
| GmWMC119 | HAKUBI | | Landrace | China |
| GmWMC120 | U 1416 | | Landrace | Nepal |
| GmJMC121 | KOSA MAME | | Landrace | Japan |
| GmWMC122 | GAPSANJAELAE(I) | | Landrace | Rep.Korea |
| GmWMC123 | N 2295 | | Landrace | Nepal |
| GmWMC124 | GU TIAN DOW | | Landrace | China |
| GmWMC125 | BHATMAS | | Landrace | Nepal |
| GmJMC126 | KOKUBU 7 | | Landrace | Japan |
| GmWMC127 | GREEN PE POKE | | Landrace | Myanmar |
| GmJMC128 | GIN DAIZU | | Landrace | Japan |
| GmWMC129 | AOKI MAME | | Landrace | China |
| GmJMC130 | HITASHIMAME | | Landrace | Japan |
| GmJMC131 | COL/EHIME/1983/UTSUNOMIYA 28 | | Landrace | Japan |
| GmWMC132 | L 2A | | Landrace | Philippines |
| GmJMC133 | DAIZU | | Landrace | Japan |
| GmWMC134 | PI 549018(ZYD3939) | | Landrace | China |
| GmWMC135 | DAU TUONG.QUOC PHONG | | Landrace | Viet Nam |
| GmWMC136 | LOCAL VAR(SEPUTIH RAMAN) | | Landrace | Indonesia |
| GmJMC137 | COL/EHIME/1983/UTSUNOMIYA 37 | | Landrace | Japan |
| GmWMC138 | COL/PAK/1989/IBPGR/2326(1) | | Landrace | Pakistan |
| GmJMC139 | BUNSEI | | Landrace | Japan |
| GmWMC140 | COL/LAOS/2005/NIAS/CED2005L25 | | Landrace | Laos |
| GmWMC141 | PETEK | | Landrace | Indonesia |
| GmWMC142 | JAVA 5 | | Landrace | Indonesia |
| GmWMC143 | M 44 | | Landrace | India |
| GmWMC144 | M 918 | | Landrace | India |
| GmJMC145 | SHIMOTSURA | | Landrace | Japan |
| GmWMC146 | HM 39 | | Landrace | India |
| GmWMC147 | COL/THAI/1986/THAI-78 | | Landrace | Thailand |
| GmWMC148 | M 42 | | Landrace | India |
| GmJMC149 | MOCHI-DAIZU | | Landrace | Japan |
| GmWMC150 | U 1042-1 | | Landrace | Nepal |
| GmWMC151 | JAVA 7 | | Landrace | Indonesia |
| GmWMC152 | U 1290-1 | | Landrace | Nepal |
| GmWMC153 | DAU NANH | | Landrace | Viet Nam |
| GmWMC154 | MANSHUU MASSHOKUTOU | | Landrace | China |
| GmWMC155 | PE POKE AHEY | | Landrace | Myanmar |
| GmWMC156 | U 8006-3 | | Landrace | Nepal |
| GmWMC157 | COL/MYANMAR/2006/U_TSUKUBA/073 | | Landrace | Myanmar |
| GmJMC158 | KUMAJI 1 | | Landrace | Japan |
| GmWMC159 | COL/PAK/1989/IBPGR/2323(2) | | Landrace | Pakistan |
| GmWMC160 | N 2392 | | Landrace | Nepal |
| GmJMC161 | ITSUKI ZAIRAI 83H | | Landrace | Japan |
| GmWMC162 | COL/THAI/1986/THAI-80 | | Landrace | Thailand |
| GmWMC163 | N 2491 | | Landrace | Nepal |
| GmWMC164 | SHAO TONG QI YUE HUANG | | Landrace | China |
| GmWMC165 | KARASUMAME(SHINCHIKU) | | Landrace | Taiwan |
| GmWMC166 | MERAPI | | Breeding line | Indonesia |
| GmJMC167 | NANKAN ZAIRAI 83 | | Landrace | Japan |
| GmWMC168 | L 317 | | Landrace | India |
| GmWMC169 | HAKUCHIKOU | | Landrace | China |
| GmWMC170 | M 652 | | Landrace | India |
| GmWMC171 | U-1741-2-2 NO.3 | | Landrace | Nepal |
| GmJMC172 | TSURUSENGOKU | | Breeding line | Japan |
| GmWMC173 | KARASUMAME(NAIHOU) | | Landrace | Taiwan |
| GmWMC174 | COL/EAST TIMOR/2005/NIAS/CED2005ET-19 | | Landrace | East Timor |
| GmWMC175 | BISHUU DAIZU | | Landrace | China |
| GmWMC176 | SANDEK SIENG | | Landrace | Cambodia |
| GmJMC177 | HAI MAME | | Landrace | Japan |
| GmWMC178 | BAO SHAN LU PI DAU | | Landrace | China |
| GmJMC179 | SAGA ZAIRAI | | Landrace | Japan |
| GmJMC180 | KOMUTA | | Landrace | Japan |
| GmWMC181 | CHIENGMAI PALMETTO | | Landrace | Thailand |
| GmWMC182 | LOCAL VAR.(TEGINENENG) | | Landrace | Indonesia |
| GmWMC183 | KARASUMAME(HEITOU) | | Landrace | Taiwan |
| GmJMC184 | BAN KURO DAIZU | | Landrace | Japan |
| GmWMC185 | JI AN HUANG DOW | | Landrace | China |
| GmWMC186 | RINGGIT | | Breeding line | Indonesia |
| GmWMC187 | KADI BHATTO | | Landrace | Nepal |
| GmWMC188 | E C 112828 | | Landrace | India |
| GmWMC189 | COL/EAST TIMOR/2005/NIAS/CED2005ET-37-2 | | Landrace | East Timor |
| GmWMC190 | SAN SAI | | Landrace | Thailand |
| GmWMC191 | MISS 33 DIXI | | Landrace | Philippines |
| GmWMC192 | U 1155-4 | | Landrace | Nepal |
| C0394 | Houjaku Kuwazu | | Landrace | Japan |
| C1322 | PK_73-54 | | Landrace | India |
| C1329 | L_323 | | Landrace | India |
| 5002T | 5002T | | Breeding line | USA |
| UA-4805 | UA-4805 | | Breeding line | USA |
| Houjaku Kuwazu | Houjaku Kuwazu | | Landrace | USA (originated from Japan) |
| Tachiutaka | Tachiutaka | | Landrace | Japan |
| C1290 | U_1176-1 | | Landrace | Nepal |

WMC: World mini-core collection; JMC: Japanese mini-core collection

**Supplementary Table 2**. Details of fertilization used in the study during each year

| Fertilizer commercial name | Applied amount | Ingredients (%) | Estimated applied amount of single element | Fertilizer Information |
| --- | --- | --- | --- | --- |
| Kumiai 366 | 12 kg/10 are | Total nitrogen 11.2  Citric soluble phosphoric acid 17.8  Water soluble phosphoric acid 13.0  Citric soluble potassium 17.8  Water soluble potassium 15.9 | N: 1.34 g m^-2^  P: 1.18 g m^-2^  K: 4.04 g m^-2^ | https://www.maff.go.jp/j/syouan/nouan/kome/k_hiryo/h20_kokuzi/attach/pdf/29hiryou-24.pdf |
| Hitachi A | 8 kg/10 are | Citric Soluble Magnesium 14.0  Water Soluble Magnesium 14.0  Water Soluble Manganese 0.30  Water Soluble Boron 0.30 | Mg: 2.24 g m^-2^  Mn: 0.024 g m^-2^  B: 0.024 g m^-2^ | https://www.zennoh.or.jp/operation/hiryou/sds/corporate/pdf/marukisyouten-2.pdf |
| Field calcium | 2.5 kg/10 are | CaO 28.5 (water-soluble 26.3)  S 17.0  pH 5.1 | Ca: 1.66 g m^-2^  S: 0.425 g m^-2^ | http://www.nankyu-c.co.jp/custom.html |

**Supplementary Table 3**. Detailed timeline of experiment and weather across 3 years

| Year |  | Temp.avg  (^o^C) | Temp.min  (^o^C) | Temp.max  (^o^C) | Radiation  (MJ) | Sowing date | Root sampling date |
| --- | --- | --- | --- | --- | --- | --- | --- |
| 2017 | Mean | 18.91 | 13.81 | 24.18 | 21.80 | May 15th | June 19-20th |
|  | SD | 2.24 | 2.57 | 3.28 | 7.64 |  |  |
|  |  |  |  |  |  |  |  |
| 2019 | Mean | 20.33 | 15.48 | 25.50 | 19.45 | May 14th | June 18-19th |
|  | SD | 1.95 | 2.19 | 3.16 | 8.61 |  |  |
|  |  |  |  |  |  |  |  |
| 2020 | Mean | 25.33 | 22.18 | 29.27 | NA | Jul 7th | Aug 9th |
|  | SD | 2.46 | 2.15 | 3.41 | NA |  |  |

**Supplementary Table 4**. List of 16 root and shoot traits measured in the study

| # | **Traits** | **Abbreviation** | **Unit** |
| --- | --- | --- | --- |
| 1 | Total root length | **TRL** | cm |
| 2 | Total number of root tips | **Tips** | tip/plant |
| 3 | Total root surface area | **Surface** | cm2 |
| 4 | Total root volume | **Vol** | cm3 |
| 5 | Root dry weight | **RDW** | mg |
| 6 | Length of thin root with diameter ≤ 0.4 mm | **ThinRL** | cm |
| 7 | Length of medium diameter root class from 0.4–1 mm | **MidRL** | cm |
| 8 | Length of thick roots with diameter > 1 mm | **ThickRL** | cm |
| 9 | Proportion of ThinRL in TRL | **ThinRL_rate** | - |
| 10 | Proportion of MidRL in TRL | **MidRL_rate** | - |
| 11 | Proportion of ThickRL in TRL | **ThickRL_rate** | - |
| 12 | Average root diameter | **Avd** | mm |
| 13 | Secondary lateral root density | **SLRD** | tip/cm |
| 14 | Nodule number per plant | **Nodule** | nodule/plant |
| 15 | Plant height | **PH** | cm |
| 16 | Shoot fresh weight | **SFW** | g |

**Supplementary Table 5.** Two-way ANOVA of root and shoot traits in each year

| Trait | *2017* | | | *2019* | | | *2020* | | |
| --- | --- | --- | --- | --- | --- | --- | --- | --- | --- |
|  | F value | | | F value | | | F value | | |
|  | Genotype | Treatment | GxT | Genotype | Treatment | GxT | Genotype | Treatment | GxT |
| TRL | 7.931*** | 207.427*** | 1.853*** | 11.912*** | 483.938*** | 3.308*** | 12.747*** | 24.177*** | 3.434*** |
| Tips | 6.178*** | 116.976*** | 1.571*** | 10.387*** | 121.797*** | 2.435*** | 7.027*** | 159.325*** | 2.720*** |
| Surface | 7.838*** | 167.815*** | 1.683*** | 12.919*** | 266.313*** | 2.775*** | 15.662*** | 15.694*** | 3.532*** |
| Vol | 7.731*** | 125.891*** | 1.578*** | 13.107*** | 103.299*** | 2.271*** | 15.362*** | 7.923** | 3.681*** |
| RDW | 9.261*** | 137.395*** | 1.887*** | 16.264*** | 153.002*** | 2.049*** | 24.261*** | 34.840*** | 5.058*** |
| ThinRL | 8.158*** | 229.315*** | 2.049*** | 11.282*** | 721.785*** | 3.843*** | 11.383*** | 23.554*** | 3.608*** |
| MidRL | 6.777*** | 89.205*** | 1.466*** | 11.709*** | 25.774*** | 2.017*** | 15.494*** | 23.384*** | 2.722*** |
| ThickRL | 6.496*** | 120.710*** | 1.707*** | 10.299*** | 107.195*** | 2.278*** | 16.149*** | 0.194ns | 4.347*** |
| Avd | 7.962*** | 31.144*** | 2.652*** | 8.903*** | 760.997*** | 3.736*** | 11.144*** | 38.831*** | 3.272*** |
| ThinRL_rate | 6.338*** | 20.394*** | 2.059*** | 8.225*** | 754.184*** | 3.971*** | 10.287*** | 0.778ns | 2.929*** |
| MidRL_rate | 6.062*** | 30.008*** | 2.013*** | 8.311*** | 832.020*** | 4.406*** | 9.449*** | 0.012ns | 2.792*** |
| ThickRL_rate | 5.973*** | 26.111*** | 1.865*** | 8.564*** | 0.702ns | 1.458*** | 5.718*** | 19.216*** | 2.578*** |
| SLRD | 1.377** | 15.208*** | 1.005ns | 2.518*** | 184.131*** | 1.170ns | 4.636*** | 51.244*** | 2.5004*** |
| Nodule | 3.948*** | 62.703*** | 3.014*** | 4.032*** | 10.518*** | 1.883*** | 8.398*** | 2.835ns | 3.567*** |
| PH | 7.326*** | 134.631*** | 2.344*** | 15.785*** | 60.548*** | 1.624*** | 22.230*** | 2.375ns | 3.298*** |
| SFW | 6.711*** | 297.324*** | 2.602*** | 15.645*** | 268.977*** | 2.594*** | 19.462*** | 110.741*** | 6.926*** |

Significance: *** p<0.001; ** p<0.01; * p<0.05; ns: not significant

TRL: total root length; Tips: total number of root tips; Surface: total root surface; Vol: Total root volume; RDW: root dry weight; ThinRL: length of thin root with diameter ≤ 0.4 mm, MidRL: length of mediam diameter root class from 0.4–1 mm, ThickRL: length of thick roots with diameter > 1 mm; ThinRL_rate: proportion of ThinRL in TRL; MidRL_rate: proportion of ThinRL in TRL; ThickRL_rate: Proportion of ThickRL in TRL; Avd: average root diameter; SLRD: secondary lateral root density; Nodule: nodule number per plant; PH: plant height; SFW: shoot fresh weight.

**Supplementary Table 6.** Three-way ANOVA of root and shoot traits across 3 years of the experiment.

| Trait | Source of variation (Mean square) | | | | | | |
| --- | --- | --- | --- | --- | --- | --- | --- |
|  | Genotype  (G) | %  (G+T+Y) | Treatment  (T) | %  (G+T+Y) | Year  (Y) | %  (G+T+Y) | Residual |
| **TRL** | 4872681*** | 2.3 | 128393277*** | 61.2 | 76590427*** | 36.5 | 209683 |
| **Tips** | 1701372*** | 2.2 | 42639485*** | 55.5 | 32490875*** | 42.3 | 111165 |
| **Surface** | 109594*** | 4.5 | 1682279*** | 69.5 | 630216*** | 26.0 | 4168 |
| **Vol** | 15.78*** | 6.2 | 125.65*** | 49.6 | 112.02*** | 44.2 | 0.57 |
| **RDW** | 99577*** | 5.8 | 819630*** | 47.6 | 802159*** | 46.6 | 2039 |
| **ThinRL** | 2592388*** | 1.7 | 92700512*** | 61.2 | 56278935*** | 37.1 | 118949 |
| **MidRL** | 343486*** | 7.7 | 1938936*** | 43.4 | 2189744*** | 49.0 | 14598 |
| **ThickRL** | 8848*** | 2.9 | 96584*** | 32.0 | 196677*** | 65.1 | 447 |
| **Avd** | 0.01*** | 0.6 | 0.296*** | 18.6 | 1.284*** | 80.8 | 0.0005 |
| **ThinRL_rate** | 0.0215*** | 1.3 | 0.7426*** | 44.6 | 0.9*** | 54.1 | 0.0015 |
| **MidRL_rate** | 0.017*** | 1.3 | 0.7425*** | 55.1 | 0.587*** | 43.6 | 0.0013 |
| **ThickRL_rate** | 0.0068*** | 15.9 | 0.0001ns | 0.2 | 0.0358*** | 83.8 | 0.00004 |
| **SLRD** | 2.1*** | 0.3 | 113*** | 13.9 | 698*** | 85.8 | 0.9 |
| **Nodule** | 713*** | 4.3 | 4680*** | 28.5 | 11056*** | 67.2 | 111 |
| **PH** | 52.3*** | 2.1 | 198*** | 7.9 | 2248*** | 90.0 | 1.6 |
| **SFW** | 30.294*** | 1.2 | 617.67*** | 24.4 | 1880.8*** | 74.4 | 4.144 |

Significance: *** p<0.001; ** p<0.01; * p<0.05; ns: not significant (F-test).

TRL: total root length; Tips: total number of root tips; Surface: total root surface; Vol: Total root volume; RDW: root dry weight; ThinRL: length of thin root with diameter ≤ 0.4 mm, MidRL: length of mediam diameter root class from 0.4–1 mm, ThickRL: length of thick roots with diameter > 1 mm; ThinRL_rate: proportion of ThinRL in TRL; MidRL_rate: proportion of ThinRL in TRL; ThickRL_rate: Proportion of ThickRL in TRL; Avd: average root diameter; SLRD: secondary lateral root density; Nodule: nodule number per plant; PH: plant height; SFW: shoot fresh weight.

| # | Trait | Factor | Xo | Xs | SD | SDperc | h2 | SG |
| --- | --- | --- | --- | --- | --- | --- | --- | --- |
| 1 | SFW | FA 1 | 4.402833 | 5.755222 | 1.352389 | 30.71633 | 0.658294 | 0.890269 |
| 2 | DW | FA 1 | 238.7936 | 305.9944 | 67.20083 | 28.14181 | 0.780689 | 52.46297 |
| 3 | TRL | FA 1 | 2112.9 | 2605.052 | 492.152 | 23.29272 | 0.765979 | 376.9779 |
| 4 | Tips | FA 1 | 1469.408 | 1774.011 | 304.6029 | 20.72963 | 0.688614 | 209.7538 |
| 5 | Surface | FA 1 | 284.7915 | 362.1138 | 77.32227 | 27.15048 | 0.7896 | 61.05363 |
| 6 | Vol | FA 1 | 2.998304 | 3.9314 | 0.933095 | 31.12076 | 0.796774 | 0.743466 |
| 7 | ThinRL | FA 1 | 1584.956 | 1926.739 | 341.783 | 21.5642 | 0.749014 | 256.0004 |
| 8 | MidRL | FA 1 | 481.6134 | 609.0652 | 127.4519 | 26.46352 | 0.788307 | 100.4712 |
| 9 | ThickRL | FA 1 | 46.29005 | 69.19948 | 22.90943 | 49.49104 | 0.693554 | 15.88893 |
| 10 | ThickRL_rate | FA 1 | 0.019922 | 0.024617 | 0.004695 | 23.56529 | 0.696316 | 0.003269 |
| 11 | Avd | FA 2 | 0.408607 | 0.420984 | 0.012377 | 3.029047 | 0.757381 | 0.009374 |
| 12 | ThinRL_rate | FA 2 | 0.749787 | 0.740488 | -0.0093 | -1.2403 | 0.692768 | -0.00644 |
| 13 | MidRL_rate | FA 2 | 0.230272 | 0.234879 | 0.004608 | 2.000903 | 0.645946 | 0.002976 |
| 14 | PH | FA 3 | 8.927778 | 10.34028 | 1.4125 | 15.82141 | 0.792892 | 1.11996 |
| 15 | SLRD | FA 3 | 3.665164 | 4.025014 | 0.359849 | 9.818097 | 0.471129 | 0.169536 |
| 16 | Nodule | FA 3 | 13.21889 | 22.81111 | 9.592222 | 72.56451 | 0.180822 | 1.734484 |

**Supplementary Table 7**. Indices for simultaneous selection of genotypes under irrigated condition

Xo: mean phenotype across panel; Xs: mean phenotype in selected genotypes; SD & SDperc: the selection differential and selection differential in percentage, respectively; h2: broad-sense heritability; SG: the selection gains.

**Supplementary Table 8**. Indices for simultaneous selection of genotypes under the non-irrigated condition

| # | Trait | Factor | Xo | Xs | SD | SDperc | h2 | SG |
| --- | --- | --- | --- | --- | --- | --- | --- | --- |
| 1 | SFW | FA 1 | 3.435703 | 5.347222 | 1.911519 | 55.63693 | 0.672458 | 1.285416 |
| 2 | PH | FA 1 | 8.420556 | 10.90556 | 2.485 | 29.51112 | 0.788138 | 1.958524 |
| 3 | DW | FA 1 | 206.8954 | 306.3694 | 99.47403 | 48.07938 | 0.797148 | 79.29551 |
| 4 | TRL | FA 1 | 1713.185 | 2364.304 | 651.1196 | 38.00638 | 0.688767 | 448.4694 |
| 5 | Tips | FA 1 | 1241.532 | 1606.833 | 365.3011 | 29.42341 | 0.649775 | 237.3634 |
| 6 | SLRD | FA 1 | 3.303116 | 3.628224 | 0.325108 | 9.842476 | 0.364905 | 0.118634 |
| 7 | Surface | FA 1 | 238.984 | 340.8149 | 101.8309 | 42.60994 | 0.743569 | 75.71835 |
| 8 | Vol | FA 1 | 2.602448 | 3.826295 | 1.223847 | 47.02677 | 0.778341 | 0.95257 |
| 9 | ThinRL | FA 1 | 1245.582 | 1696.484 | 450.9019 | 36.2001 | 0.645478 | 291.0474 |
| 10 | MidRL | FA 1 | 431.7991 | 604.2965 | 172.4974 | 39.94853 | 0.751358 | 129.6073 |
| 11 | ThickRL | FA 1 | 35.75576 | 63.4744 | 27.71863 | 77.52214 | 0.705754 | 19.56254 |
| 12 | ThickRL_rate | FA 1 | 0.020056 | 0.026596 | 0.00654 | 32.61094 | 0.721772 | 0.004721 |
| 13 | Nodule | FA 1 | 10.75569 | 17.24722 | 6.491528 | 60.35433 | 0.408988 | 2.654955 |
| 14 | Avd | FA 2 | 0.427989 | 0.439688 | 0.011698 | 2.733311 | 0.626521 | 0.007329 |
| 15 | ThinRL_rate | FA 2 | 0.720077 | 0.714594 | -0.00548 | -0.7614 | 0.520763 | -0.00286 |
| 16 | MidRL_rate | FA 2 | 0.259845 | 0.25879 | -0.00106 | -0.40602 | 0.476102 | -0.0005 |

Xo: mean phenotype across panel; Xs: mean phenotype in selected genotypes; SD & SDperc: the selection differential and selection differential in percentage, respectively; h2: broad-sense heritability; SG: the selection gains.

**Supplementary Table 9**. Rank of genotypes based on MTSI scores under irrigated and non-irrigated conditions

| **Irrigated** | | |  | **Non-irrigated** | | |
| --- | --- | --- | --- | --- | --- | --- |
| Rank | Genotype | MTSI |  | Rank | Genotype | MTSI |
| 1 | GmWMC072 | 6.081143 |  | 1 | GmJMC068 | 3.26336 |
| 2 | GmJMC105 | 6.187983 |  | 2 | GmJMC092 | 3.280981 |
| 3 | GmWMC148 | 6.280022 |  | 3 | GmJMC033 | 3.426665 |
| 4 | GmJMC130 | 6.351185 |  | 4 | GmJMC016 | 3.501734 |
| 5 | GmWMC029 | 6.361234 |  | 5 | GmJMC102 | 3.54108 |
| 6 | GmWMC178 | 6.368441 |  | 6 | GmJMC130 | 3.589874 |
| 7 | GmJMC092 | 6.389233 |  | 7 | GmWMC075 | 3.618732 |
| 8 | GmWMC123 | 6.574063 |  | 8 | GmJMC093 | 3.66487 |
| 9 | GmJMC099 | 6.615783 |  | 9 | GmWMC094 | 3.707057 |
| 10 | GmJMC110 | 6.643155 |  | 10 | GmWMC152 | 3.708929 |
| 11 | GmJMC091 | 6.690953 |  | 11 | GmJMC025 | 3.72647 |
| 12 | GmJMC068 | 6.699468 |  | 12 | GmJMC085 | 3.765789 |
| 13 | GmWMC153 | 6.726321 |  | 13 | GmJMC111 | 3.803505 |
| 14 | GmWMC066 | 6.862191 |  | 14 | GmWMC178 | 3.874797 |
| 15 | GmWMC175 | 6.875843 |  | 15 | GmWMC147 | 3.972723 |
| 16 | GmJMC058 | 6.928193 |  | 16 | GmJMC054 | 4.008115 |
| 17 | GmWMC075 | 6.98278 |  | 17 | GmJMC081 | 4.011993 |
| 18 | GmWMC086 | 6.991641 |  | 18 | GmJMC088 | 4.075141 |
| 19 | GmJMC059 | 7.018529 |  | 19 | GmJMC112 | 4.081647 |
| 20 | GmJMC081 | 7.03 |  | 20 | GmJMC056 | 4.10651 |
| 21 | GmJMC067 | 7.046071 |  | 21 | GmJMC104 | 4.111077 |
| 22 | GmJMC177 | 7.143227 |  | 22 | GmJMC099 | 4.163877 |
| 23 | GmJMC017 | 7.154004 |  | 23 | GmJMC065 | 4.164929 |
| 24 | GmWMC143 | 7.199083 |  | 24 | GmJMC059 | 4.205563 |
| 25 | GmJMC078 | 7.206855 |  | 25 | GmJMC179 | 4.223572 |
| 26 | GmJMC028 | 7.265539 |  | 26 | GmJMC021 | 4.257963 |
| 27 | GmWMC012 | 7.293366 |  | 27 | GmWMC010 | 4.269334 |
| 28 | GmWMC094 | 7.303011 |  | 28 | GmWMC123 | 4.284111 |
| 29 | GmJMC021 | 7.316649 |  | 29 | GmJMC055 | 4.313998 |
| 30 | GmWMC147 | 7.32278 |  | 30 | GmJMC101 | 4.342388 |
| 31 | GmWMC125 | 7.369755 |  | 31 | GmJMC082 | 4.360087 |
| 32 | GmJMC104 | 7.421329 |  | 32 | GmWMC135 | 4.373682 |
| 33 | GmJMC041 | 7.475595 |  | 33 | GmJMC091 | 4.385829 |
| 34 | GmJMC100 | 7.478284 |  | 34 | GmWMC109 | 4.417399 |
| 35 | GmJMC096 | 7.50198 |  | 35 | GmWMC125 | 4.421165 |
| 36 | GmJMC050 | 7.528747 |  | 36 | GmJMC060 | 4.421296 |
| 37 | GmJMC032 | 7.529111 |  | 37 | GmWMC185 | 4.442916 |
| 38 | GmJMC085 | 7.551689 |  | 38 | C0394 | 4.444044 |
| 39 | GmJMC082 | 7.55298 |  | 39 | GmWMC164 | 4.446978 |
| 40 | GmWMC011 | 7.573892 |  | 40 | GmJMC023 | 4.450892 |
| 41 | GmWMC109 | 7.616615 |  | 41 | GmJMC031 | 4.458808 |
| 42 | GmWMC048 | 7.635827 |  | 42 | GmJMC139 | 4.470691 |
| 43 | GmWMC035 | 7.644849 |  | 43 | GmJMC078 | 4.470833 |
| 44 | GmJMC167 | 7.657182 |  | 44 | GmJMC062 | 4.474036 |
| 45 | GmWMC122 | 7.663046 |  | 45 | GmWMC066 | 4.491791 |
| 46 | GmJMC008 | 7.677283 |  | 46 | GmJMC105 | 4.492943 |
| 47 | GmWMC185 | 7.697748 |  | 47 | GmJMC079 | 4.496005 |
| 48 | GmJMC101 | 7.700318 |  | 48 | GmWMC011 | 4.496321 |
| 49 | HOUJAKU_KUWAZU | 7.701866 |  | 49 | GmJMC067 | 4.500259 |
| 50 | GmWMC187 | 7.702823 |  | 50 | C1322 | 4.524112 |
| 51 | GmJMC131 | 7.740667 |  | 51 | GmJMC114 | 4.546369 |
| 52 | GmJMC016 | 7.74997 |  | 52 | GmWMC006 | 4.546755 |
| 53 | GmJMC060 | 7.751179 |  | 53 | GmJMC039 | 4.55595 |
| 54 | GmJMC049 | 7.757777 |  | 54 | GmJMC069 | 4.56212 |
| 55 | GmJMC031 | 7.76559 |  | 55 | GmWMC018 | 4.564152 |
| 56 | GmWMC071 | 7.793688 |  | 56 | GmWMC175 | 4.565079 |
| 57 | GmJMC076 | 7.796743 |  | 57 | GmWMC071 | 4.595104 |
| 58 | GmWMC181 | 7.808988 |  | 58 | GmWMC148 | 4.607093 |
| 59 | GmWMC118 | 7.809577 |  | 59 | GmJMC064 | 4.627586 |
| 60 | GmJMC003 | 7.811135 |  | 60 | GmJMC004 | 4.629453 |
| 61 | GmJMC007 | 7.821546 |  | 61 | GmJMC007 | 4.629707 |
| 62 | C1322 | 7.831034 |  | 62 | GmJMC050 | 4.631038 |
| 63 | GmJMC088 | 7.83807 |  | 63 | GmJMC161 | 4.637942 |
| 64 | GmWMC018 | 7.852796 |  | 64 | GmJMC077 | 4.656736 |
| 65 | GmJMC009 | 7.871584 |  | 65 | GmWMC027 | 4.657841 |
| 66 | GmWMC152 | 7.879965 |  | 66 | GmWMC029 | 4.681499 |
| 67 | GmJMC051 | 7.916281 |  | 67 | GmJMC076 | 4.691147 |
| 68 | GmWMC074 | 7.923899 |  | 68 | GmJMC110 | 4.695393 |
| 69 | GmJMC002 | 7.931932 |  | 69 | GmJMC041 | 4.715806 |
| 70 | GmJMC093 | 7.938997 |  | 70 | GmJMC095 | 4.744417 |
| 71 | GmWMC001 | 7.980532 |  | 71 | GmJMC044 | 4.746643 |
| 72 | GmJMC111 | 7.981527 |  | 72 | GmWMC070 | 4.767025 |
| 73 | GmJMC053 | 7.982175 |  | 73 | GmJMC005 | 4.77835 |
| 74 | GmJMC052 | 7.98669 |  | 74 | GmWMC048 | 4.785344 |
| 75 | GmWMC027 | 7.987861 |  | 75 | GmJMC040 | 4.794272 |
| 76 | GmWMC163 | 8.000813 |  | 76 | GmWMC124 | 4.795264 |
| 77 | GmJMC055 | 8.002213 |  | 77 | GmJMC037 | 4.796476 |
| 78 | GmJMC039 | 8.034275 |  | 78 | GmJMC128 | 4.809586 |
| 79 | C0394 | 8.045126 |  | 79 | GmJMC149 | 4.81585 |
| 80 | GmJMC037 | 8.053605 |  | 80 | GmJMC051 | 4.823219 |
| 81 | GmJMC080 | 8.05651 |  | 81 | GmWMC089 | 4.827862 |
| 82 | GmJMC057 | 8.058448 |  | 82 | GmJMC063 | 4.845398 |
| 83 | GmJMC025 | 8.059189 |  | 83 | GmWMC169 | 4.864879 |
| 84 | GmWMC127 | 8.078354 |  | 84 | GmWMC014 | 4.881383 |
| 85 | GmJMC005 | 8.094725 |  | 85 | GmJMC126 | 4.920496 |
| 86 | GmWMC136 | 8.104336 |  | 86 | GmJMC131 | 4.930268 |
| 87 | GmJMC033 | 8.112758 |  | 87 | GmJMC017 | 4.940024 |
| 88 | GmJMC064 | 8.116972 |  | 88 | GmWMC129 | 5.012428 |
| 89 | GmWMC164 | 8.121046 |  | 89 | GmWMC073 | 5.015881 |
| 90 | GmWMC087 | 8.133765 |  | 90 | GmJMC097 | 5.034839 |
| 91 | GmWMC124 | 8.169161 |  | 91 | GmWMC119 | 5.046931 |
| 92 | GmJMC062 | 8.170287 |  | 92 | GmJMC167 | 5.058858 |
| 93 | C1290 | 8.186496 |  | 93 | GmJMC026 | 5.067236 |
| 94 | Williams82 | 8.190357 |  | 94 | GmJMC061 | 5.071538 |
| 95 | GmJMC114 | 8.21594 |  | 95 | GmWMC103 | 5.086769 |
| 96 | GmJMC065 | 8.216072 |  | 96 | GmJMC028 | 5.091486 |
| 97 | GmJMC149 | 8.238249 |  | 97 | GmJMC080 | 5.129722 |
| 98 | GmWMC138 | 8.24378 |  | 98 | GmWMC107 | 5.148182 |
| 99 | GmWMC089 | 8.272972 |  | 99 | GmJMC030 | 5.217009 |
| 100 | GmWMC120 | 8.283666 |  | 100 | GmWMC132 | 5.250329 |
| 101 | GmWMC169 | 8.299641 |  | 101 | GmJMC009 | 5.251112 |
| 102 | GmWMC119 | 8.302364 |  | 102 | GmJMC090 | 5.303397 |
| 103 | GmWMC046 | 8.311204 |  | 103 | HOUJAKU_KUWAZU | 5.319458 |
| 104 | GmJMC034 | 8.340189 |  | 104 | Tachiutaka | 5.323216 |
| 105 | GmJMC117 | 8.360262 |  | 105 | GmWMC181 | 5.326365 |
| 106 | GmJMC139 | 8.366688 |  | 106 | Williams82 | 5.333965 |
| 107 | GmJMC116 | 8.374103 |  | 107 | GmJMC116 | 5.359195 |
| 108 | GmJMC054 | 8.378208 |  | 108 | GmJMC137 | 5.396038 |
| 109 | GmJMC069 | 8.400175 |  | 109 | GmWMC045 | 5.40576 |
| 110 | GmJMC043 | 8.420221 |  | 110 | GmJMC003 | 5.428213 |
| 111 | GmJMC026 | 8.421297 |  | 111 | GmWMC171 | 5.429682 |
| 112 | GmWMC171 | 8.434695 |  | 112 | GmJMC049 | 5.432193 |
| 113 | GmWMC155 | 8.471251 |  | 113 | GmWMC074 | 5.438226 |
| 114 | GmJMC095 | 8.491695 |  | 114 | GmJMC043 | 5.441409 |
| 115 | GmJMC121 | 8.494175 |  | 115 | GmWMC122 | 5.480801 |
| 116 | GmJMC044 | 8.499303 |  | 116 | GmJMC133 | 5.490407 |
| 117 | GmWMC135 | 8.51925 |  | 117 | GmJMC098 | 5.498653 |
| 118 | GmWMC010 | 8.52858 |  | 118 | GmWMC086 | 5.507992 |
| 119 | GmJMC098 | 8.533331 |  | 119 | GmJMC121 | 5.518618 |
| 120 | GmWMC151 | 8.540728 |  | 120 | GmJMC047 | 5.520003 |
| 121 | GmWMC160 | 8.554866 |  | 121 | GmJMC184 | 5.5232 |
| 122 | GmJMC179 | 8.56657 |  | 122 | GmJMC106 | 5.538166 |
| 123 | GmWMC192 | 8.566649 |  | 123 | GmJMC008 | 5.552908 |
| 124 | GmWMC070 | 8.573135 |  | 124 | GmJMC053 | 5.578336 |
| 125 | GmJMC145 | 8.62224 |  | 125 | GmJMC052 | 5.587357 |
| 126 | GmJMC030 | 8.626111 |  | 126 | GmWMC189 | 5.591941 |
| 127 | GmJMC077 | 8.637012 |  | 127 | GmJMC117 | 5.59538 |
| 128 | GmWMC113 | 8.65656 |  | 128 | GmWMC020 | 5.611473 |
| 129 | GmWMC107 | 8.65824 |  | 129 | GmWMC118 | 5.647807 |
| 130 | GmWMC132 | 8.660014 |  | 130 | GmWMC113 | 5.726116 |
| 131 | GmJMC079 | 8.698278 |  | 131 | GmWMC001 | 5.740941 |
| 132 | Tachiutaka | 8.698786 |  | 132 | GmWMC141 | 5.744156 |
| 133 | GmWMC168 | 8.707316 |  | 133 | GmWMC143 | 5.761292 |
| 134 | GmWMC182 | 8.740667 |  | 134 | GmJMC177 | 5.777713 |
| 135 | GmJMC063 | 8.74342 |  | 135 | GmJMC057 | 5.805652 |
| 136 | GmWMC144 | 8.77351 |  | 136 | 5002T | 5.816908 |
| 137 | GmWMC165 | 8.774921 |  | 137 | GmWMC087 | 5.828946 |
| 138 | GmWMC015 | 8.794933 |  | 138 | GmWMC046 | 5.832645 |
| 139 | GmJMC061 | 8.798805 |  | 139 | UA-4805 | 5.839528 |
| 140 | GmJMC133 | 8.833742 |  | 140 | GmWMC038 | 5.851493 |
| 141 | GmWMC154 | 8.848929 |  | 141 | GmWMC120 | 5.859889 |
| 142 | GmJMC126 | 8.849499 |  | 142 | GmWMC083 | 5.886518 |
| 143 | GmWMC140 | 8.857289 |  | 143 | GmWMC024 | 5.907157 |
| 144 | GmWMC006 | 8.866155 |  | 144 | GmJMC002 | 5.963317 |
| 145 | C1329 | 8.894927 |  | 145 | GmJMC180 | 6.006049 |
| 146 | GmWMC045 | 8.961244 |  | 146 | GmWMC022 | 6.0284 |
| 147 | GmJMC112 | 8.966555 |  | 147 | GmWMC188 | 6.04459 |
| 148 | GmWMC103 | 8.981523 |  | 148 | GmWMC173 | 6.05789 |
| 149 | GmWMC038 | 8.99108 |  | 149 | GmWMC187 | 6.058369 |
| 150 | 5002T | 9.014302 |  | 150 | GmWMC153 | 6.082808 |
| 151 | GmJMC172 | 9.016663 |  | 151 | GmWMC127 | 6.088409 |
| 152 | GmJMC004 | 9.045376 |  | 152 | GmWMC140 | 6.09987 |
| 153 | GmWMC189 | 9.050366 |  | 153 | GmWMC168 | 6.122749 |
| 154 | GmJMC047 | 9.082201 |  | 154 | GmWMC162 | 6.133685 |
| 155 | GmWMC156 | 9.090312 |  | 155 | GmJMC032 | 6.150475 |
| 156 | GmWMC083 | 9.094482 |  | 156 | GmWMC190 | 6.152006 |
| 157 | GmWMC019 | 9.113355 |  | 157 | GmWMC072 | 6.157437 |
| 158 | GmWMC159 | 9.132762 |  | 158 | GmWMC035 | 6.168901 |
| 159 | GmJMC023 | 9.141751 |  | 159 | GmWMC146 | 6.195466 |
| 160 | GmJMC158 | 9.158949 |  | 160 | GmWMC166 | 6.21974 |
| 161 | UA-4805 | 9.179431 |  | 161 | GmWMC170 | 6.289033 |
| 162 | GmJMC040 | 9.191015 |  | 162 | GmWMC144 | 6.339271 |
| 163 | GmJMC106 | 9.191335 |  | 163 | GmWMC015 | 6.339326 |
| 164 | GmWMC188 | 9.220548 |  | 164 | GmWMC142 | 6.348758 |
| 165 | GmWMC157 | 9.239716 |  | 165 | GmWMC012 | 6.354217 |
| 166 | GmJMC184 | 9.281295 |  | 166 | GmWMC182 | 6.365654 |
| 167 | GmWMC084 | 9.325931 |  | 167 | GmWMC154 | 6.436443 |
| 168 | GmJMC097 | 9.327152 |  | 168 | GmWMC186 | 6.493952 |
| 169 | GmJMC013 | 9.377182 |  | 169 | GmJMC158 | 6.515315 |
| 170 | GmWMC024 | 9.411503 |  | 170 | GmJMC145 | 6.5616 |
| 171 | GmWMC014 | 9.417718 |  | 171 | GmWMC160 | 6.56427 |
| 172 | GmJMC128 | 9.444522 |  | 172 | GmWMC191 | 6.596055 |
| 173 | GmJMC161 | 9.456408 |  | 173 | GmWMC151 | 6.60112 |
| 174 | GmWMC108 | 9.458846 |  | 174 | GmWMC155 | 6.603537 |
| 175 | GmJMC056 | 9.459346 |  | 175 | GmJMC096 | 6.658005 |
| 176 | GmWMC022 | 9.525959 |  | 176 | GmJMC058 | 6.671048 |
| 177 | GmWMC150 | 9.554162 |  | 177 | GmWMC036 | 6.706492 |
| 178 | GmWMC186 | 9.563467 |  | 178 | GmJMC013 | 6.726762 |
| 179 | GmWMC170 | 9.565848 |  | 179 | C1290 | 6.728197 |
| 180 | GmJMC090 | 9.573106 |  | 180 | GmJMC172 | 6.741503 |
| 181 | GmWMC073 | 9.580384 |  | 181 | GmWMC163 | 6.758635 |
| 182 | GmWMC129 | 9.584742 |  | 182 | GmWMC019 | 6.780468 |
| 183 | GmWMC190 | 9.607721 |  | 183 | GmJMC100 | 6.781374 |
| 184 | GmJMC102 | 9.653788 |  | 184 | GmWMC174 | 6.795726 |
| 185 | GmWMC183 | 9.701902 |  | 185 | GmWMC156 | 6.848245 |
| 186 | GmWMC173 | 9.862391 |  | 186 | GmWMC136 | 6.863117 |
| 187 | GmWMC191 | 9.876159 |  | 187 | GmWMC138 | 6.869773 |
| 188 | GmWMC146 | 9.955826 |  | 188 | GmWMC183 | 6.876547 |
| 189 | GmWMC042 | 10.02927 |  | 189 | GmWMC084 | 6.900296 |
| 190 | GmWMC142 | 10.04005 |  | 190 | GmWMC165 | 6.908402 |
| 191 | GmWMC166 | 10.11038 |  | 191 | C1329 | 6.990539 |
| 192 | GmWMC162 | 10.30071 |  | 192 | GmWMC042 | 7.039819 |
| 193 | GmWMC036 | 10.32233 |  | 193 | GmWMC159 | 7.203914 |
| 194 | GmWMC174 | 10.58464 |  | 194 | GmJMC034 | 7.219395 |
| 195 | GmJMC137 | 10.59638 |  | 195 | GmWMC192 | 7.272347 |
| 196 | GmWMC141 | 10.66547 |  | 196 | GmWMC157 | 7.506082 |
| 197 | GmJMC180 | 10.68066 |  | 197 | GmWMC150 | 7.610491 |
| 198 | GmWMC020 | 11.20369 |  | 198 | GmWMC134 | 7.7434 |
| 199 | GmWMC176 | 11.25402 |  | 199 | GmWMC108 | 7.829918 |
| 200 | GmWMC134 | 11.29935 |  | 200 | GmWMC176 | 8.286412 |


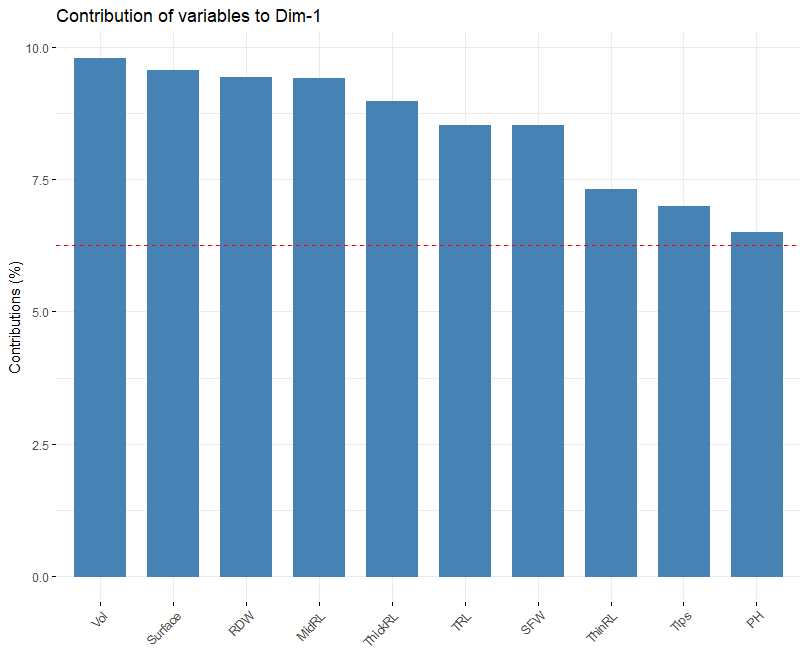

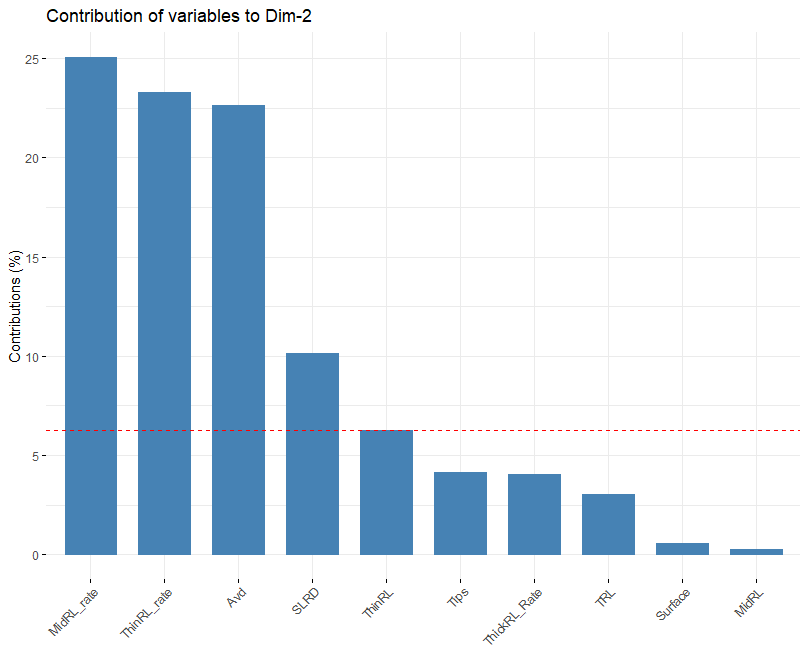


**Supplementary Figure 1**. Contribution of key traits to the two first PCs of PCA using best linear unbiased prediction (BLUP) values of 16 root and shoot traits.


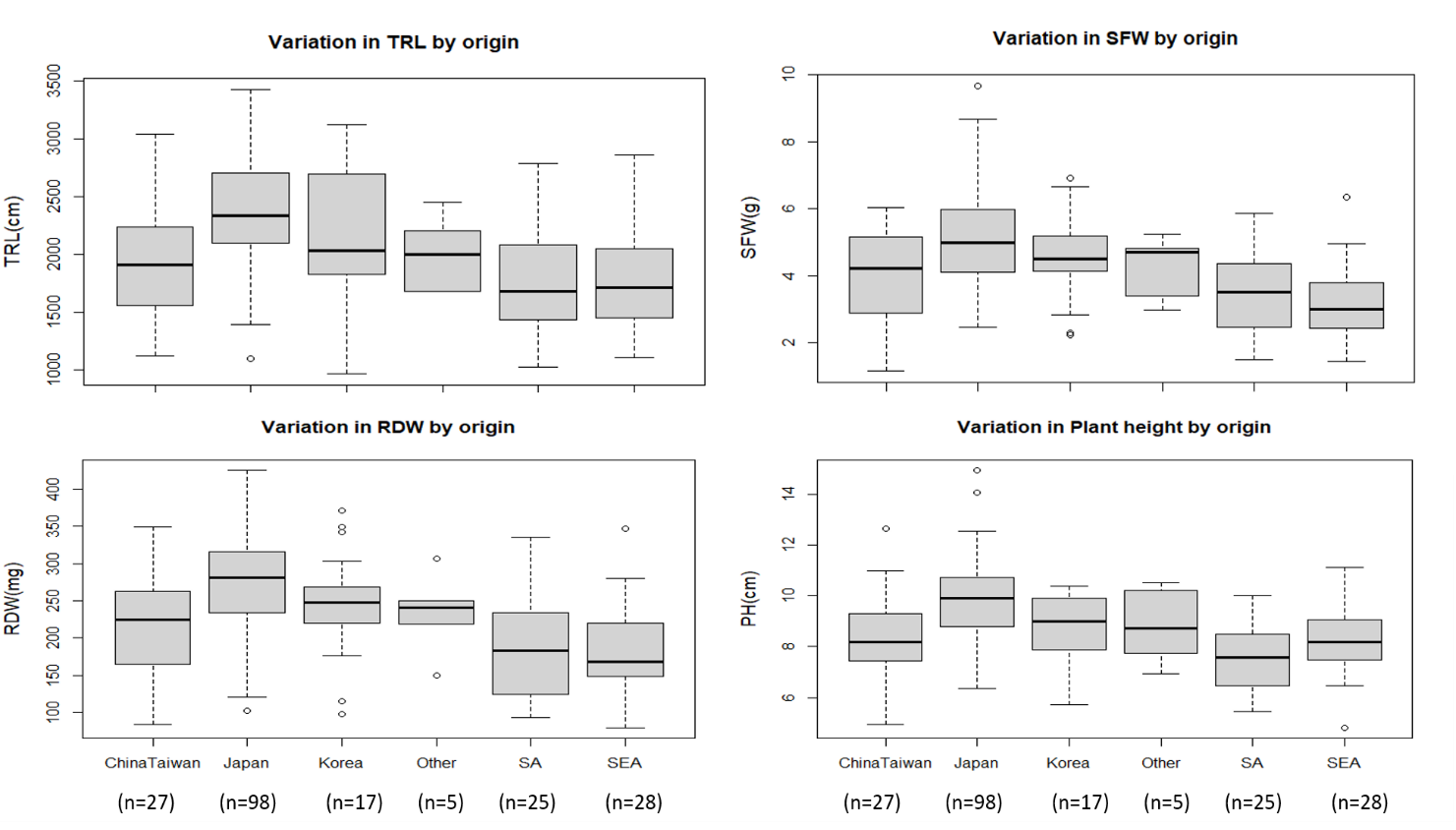


**Supplementary Figure 2.** Boxplots showing variation in total root length (TRL), root dry weight (RDW), shoot fresh weight (SFW), and plant height (PH) among genotypes from different origins using best linear unbiased prediction (BLUP) values in irrigated conditions. ChinaTaiwan: China and Taiwan; SA: South Asia (India, Pakistan, Nepal); SEA: South East Asia.


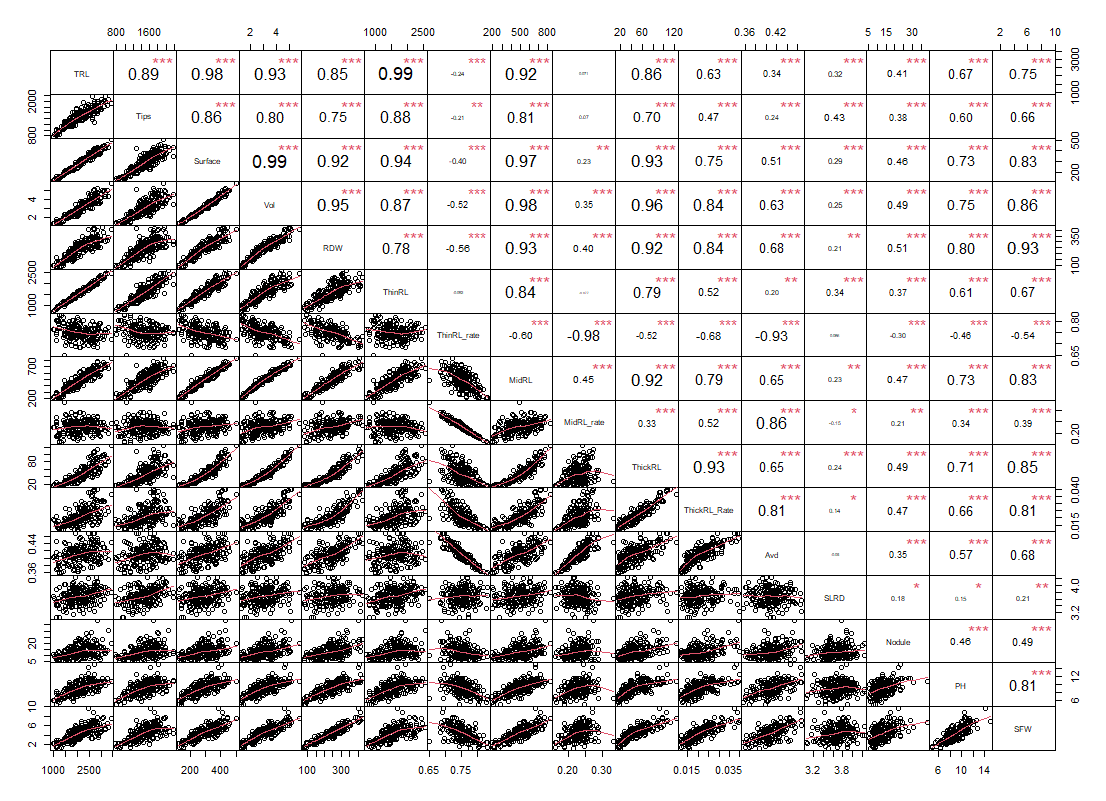


**Supplementary Figure 3**. Pearson correlations among root and shoot traits in irrigated conditions using best linear unbiased prediction (BLUP) data. TRL: total root length; Tips: total number of root tips; Surface: total root surface; Vol: Total root volume; RDW: root dry weight; ThinRL: length of thin root with diameter ≤ 0.4 mm, MidRL: length of medium diameter root class from 0.4–1 mm, ThickRL: length of thick roots with diameter >1 mm; ThinRL_rate: proportion of ThinRL in TRL; MidRL_rate: proportion of ThinRL in TRL; ThickRL_rate: Proportion of ThickRL in TRL; Avd: average root diameter; SLRD: secondary lateral root density; Nodule: nodule number per plant; PH: plant height; SFW: shoot fresh weight.


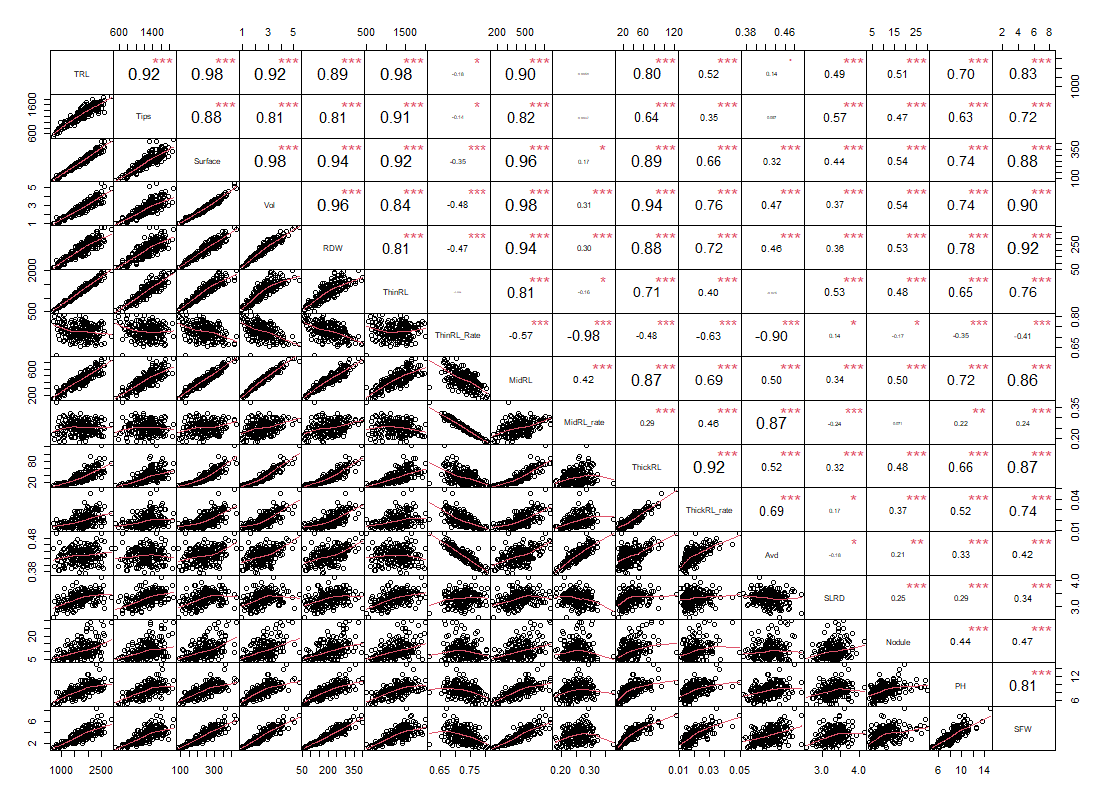


**Supplementary Figure 4**. Pearson correlations among root and shoot traits in non-irrigated conditions using best linear unbiased prediction (BLUP) data. TRL: total root length; Tips: total number of root tips; Surface: total root surface; Vol: Total root volume; RDW: root dry weight; ThinRL: length of thin root with diameter ≤ 0.4mm, MidRL: length of middle diameter root class from 0.4–1 mm, ThickRL: length of thick roots with diameter >1 mm; ThinRL_rate: proportion of ThinRL in TRL; MidRL_rate: proportion of ThinRL in TRL; ThickRL_rate: Proportion of ThickRL in TRL; Avd: average root diameter; SLRD: secondary lateral root density; Nodule: nodule number per plant; PH: plant height; SFW: shoot fresh weight.

I-index

RI-index

**Supplementary Figure 5**. Comparison between the increment (I-index) and relative increment (RI-index) indices of total root length (TRL) among 200 soybean genotypes. Range of I-index: 0.78–1.83 (237%). Range of RI-index: 0.23–2.63(1100%).


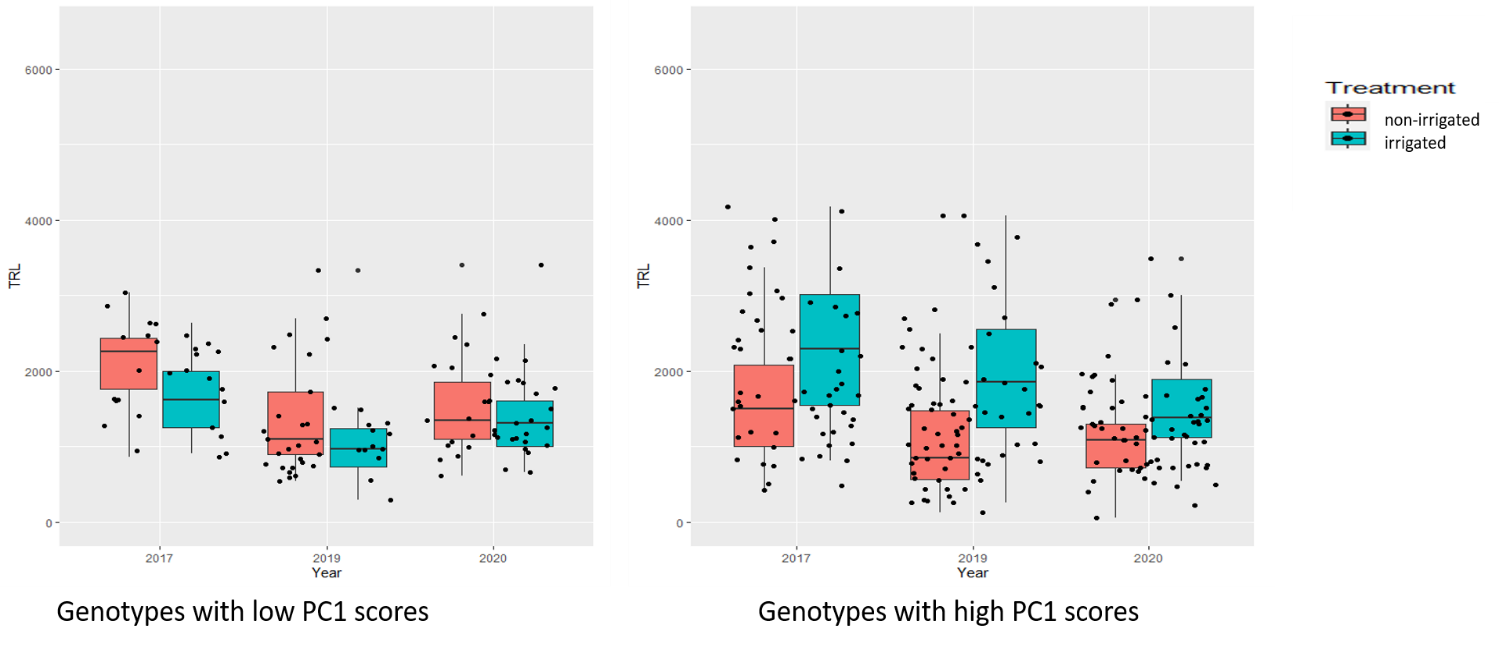


**Supplementary Figure 6**. Box plots showing original values of total root length (TRL; cm) over 3 years among two groups of selected genotypes based on PCA using the increment-index.

Genotypes with low PC1 scores: GmJMC098, GmWMC141, GmJMC079, GmWMC174, GmWMC015, GmJMC180, and GmJMC060. Genotypes with high PC1 scores: GmJMC065, GmWMC176, GmWMC150, GmJMC116, GmWMC136, GmJMC096, GmJMC013, GmWMC163, GmWMC108, GmWMC113, GmWMC118, GmWMC073, and GmWMC144.


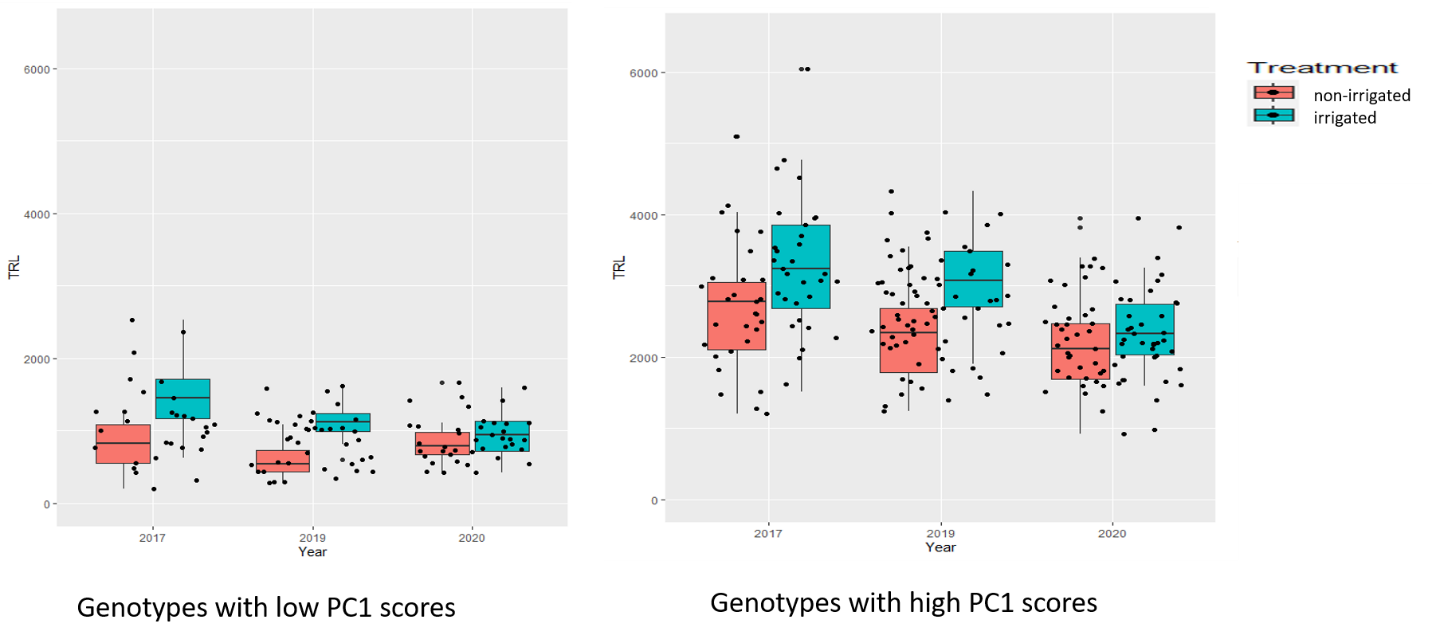


**Supplementary Figure 7**. Box plots showing original values of total root length (TRL; cm) over 3 years among two groups of selected genotypes based on PCA using the relative increment-index.

Genotypes with low PC1 scores: GmWMC108, GmWMC157, GmWMC159, GmWMC160, GmWMC176, GmWMC192. Genotypes with high PC1 scores: GmJMC033, GmJMC054, GmJMC068, GmJMC076, GmJMC077, GmJMC091, GmJMC092, GmJMC101, GmJMC102, GmJMC105, GmJMC110, GmJMC130, GmWMC042, and GmWMC066.


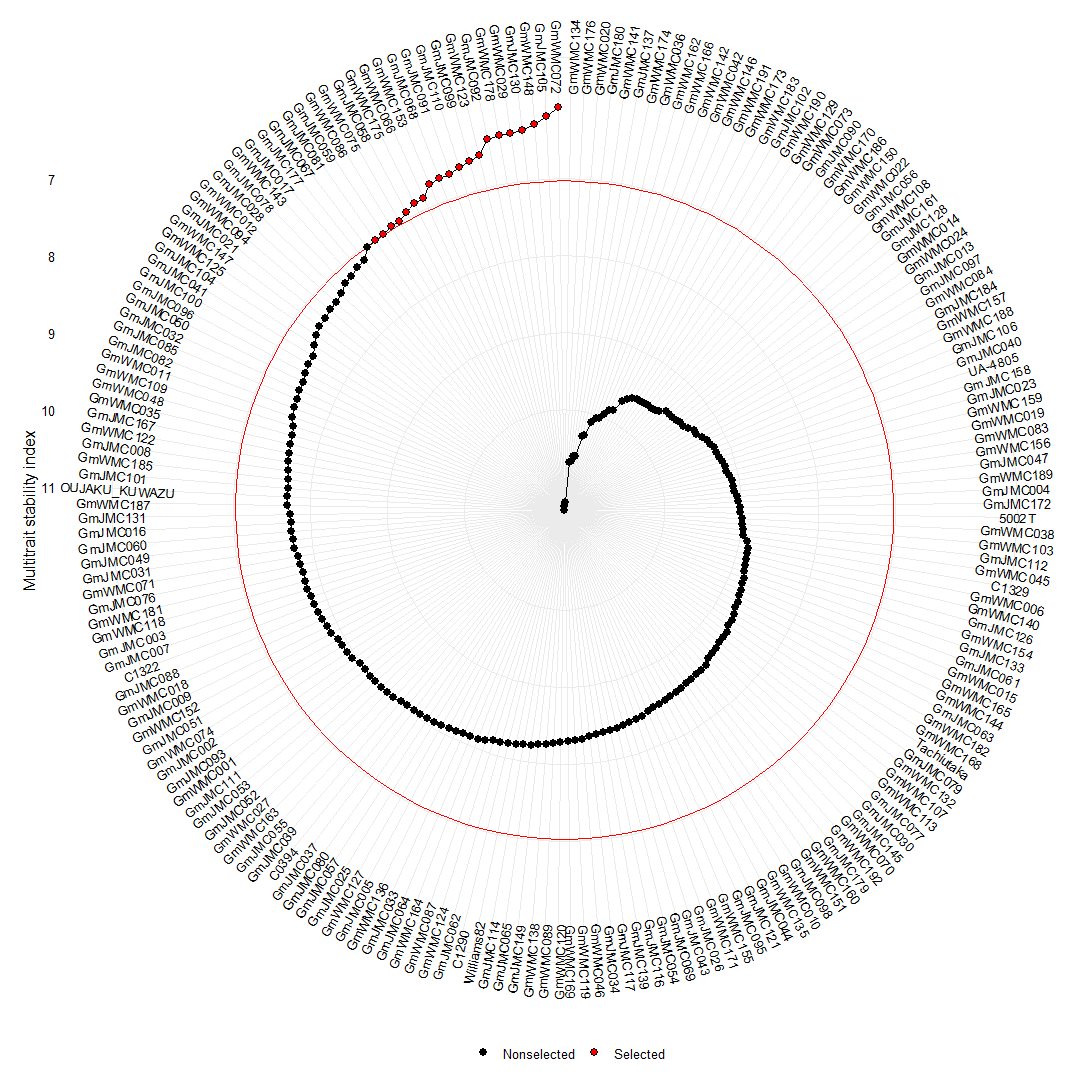


**Supplementary Figure 8.** Selection of 10% of genotypes with high mean performance and stability across 3 years under irrigated conditions using multiple trait selection index (MTSI) (weighted average of absolute scores (WAASBY) index values) using 16 root and shoot traits.


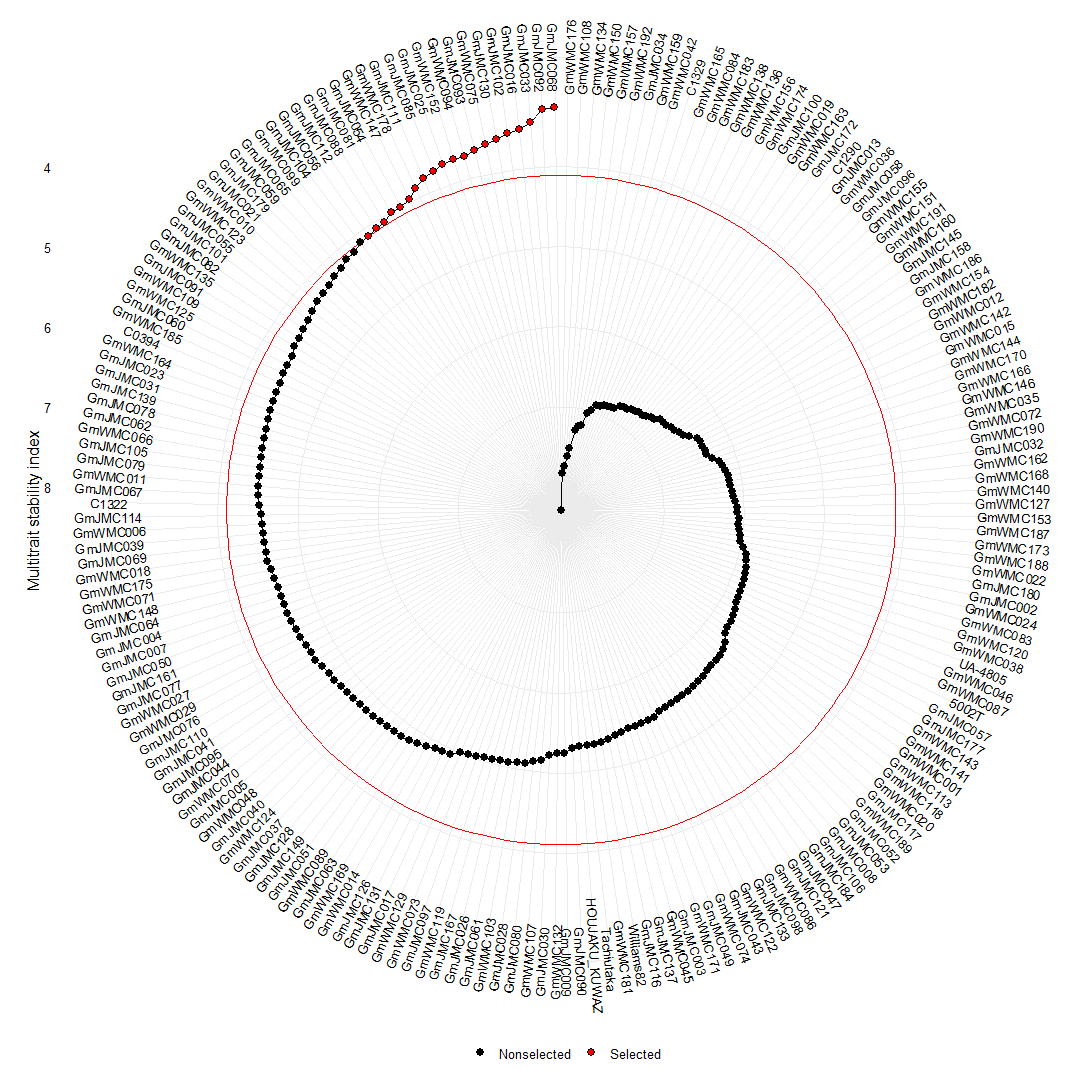


**Supplementary Figure 9.** Selection of 10% of genotypes with high mean performance and stability across 3 years under non-irrigated conditions using multiple trait selection index (MTSI) (weighted average of absolute scores (WAASBY) index values) using 16 root and shot traits.
